# Prognostic Nutritional Index (PNI) in Patients With Breast Cancer Treated With Neoadjuvant Chemotherapy as a Useful Prognostic Indicator

**DOI:** 10.3389/fcell.2021.656741

**Published:** 2021-03-30

**Authors:** Li Chen, Ping Bai, Xiangyi Kong, Shaolong Huang, Zhongzhao Wang, Xiangyu Wang, Yi Fang, Jing Wang

**Affiliations:** ^1^Department of Breast Surgical Oncology, National Cancer Center/National Clinical Research Center for Cancer/Cancer Hospital, Chinese Academy of Medical Sciences and Peking Union Medical College, Beijing, China; ^2^Department of Operation Room, National Cancer Center/National Clinical Research Center for Cancer/Cancer Hospital, Chinese Academy of Medical Sciences and Peking Union Medical College, Beijing, China; ^3^Department of Breast and Thyroid, Traumatic and Plastic Surgery, Tongren Municipal People’s Hospital, Guizhou, China

**Keywords:** prognostic nutritional index, breast cancer, neoadjuvant chemotherapy, survival, biomarker

## Abstract

**Objective:**

Prognostic nutritional index (PNI), calculated as serum albumin (ALB) (g/L) + 5 × total lymphocyte count (10^9^/L), is initially used to evaluate nutritional status in patients undergoing surgery and may evaluate the therapeutic effects and predict the survival of various solid tumors. The present study aimed to evaluate the potential prognostic significance of PNI in breast cancer patients receiving neoadjuvant chemotherapy (NACT).

**Methods:**

A total of 785 breast cancer patients treated with neoadjuvant chemotherapy were enrolled in this retrospective study. The optimal cutoff value of PNI by receiver operating characteristic curve stratified patients into a low-PNI group (<51) and a high PNI group (≥51). The associations between breast cancer and clinicopathological variables by PNI were determined by chi-square test or Fisher’s exact test. Kaplan–Meier plots and log-rank test were used to evaluate the clinical outcomes of disease-free survival (DFS) and overall survival (OS). The prognostic value of PNI was analyzed by univariate and multivariate Cox proportional hazards regression models. The toxicity of NACT was accessed by the National Cancer Institute Common Toxicity Criteria (NCI-CTC).

**Results:**

The results indicated that PNI had prognostic significance by an optimal cutoff value of 51 on DFS and OS in univariate and multivariate Cox regression survival analyses. Breast cancer patients with a high PNI value had longer DFS and OS than those with a low PNI value [47.64 *vs*. 36.60 months, *P* < 0.0001, hazard ratio (HR) = 0.264, 95%CI = 0.160–0.435; 73.61 *vs*. 64.97 months, *P* < 0.0001, HR = 0.319, 95%CI = 0.207–0.491, respectively]. Furthermore, the results indicated that patients with high PNI had longer DFS and OS than those with low PNI in early stage and advanced breast cancer, especially in advanced breast cancer. The mean DFS and OS times for breast cancer patients with high PNI by the log-rank test were longer than in those with low PNI in different molecular subtypes. Moreover, the mean DFS and OS times in patients with high PNI by the log-rank test were longer than in those patients with low PNI without or with lymph vessel invasion. The common toxicities after neoadjuvant chemotherapy were hematologic and gastrointestinal reaction, and the PNI had no significance on the toxicities of all enrolled patients, except in anemia, leukopenia, and myelosuppression.

**Conclusion:**

Pretreatment PNI with the advantages of being convenient, noninvasive, and reproducible was a useful prognostic indicator for breast cancer patients receiving neoadjuvant chemotherapy and is a promising biomarker for breast cancer on treatment strategy decisions.

## Introduction

Breast cancer is the most common cancer in women and is the most frequent cause of cancer-related morbidity and mortality for women throughout the world (Siegel et al., 2019). The incidence of breast cancer is increasing year after year, and the survivors with this diagnosis account for almost one fourth of the over 14 million cancer survivors in the United States (Ganz and Goodwin, 2015). As many basic and clinical trial research have been conducted in breast cancer for several decades, we have learned much about the mechanisms of breast cancer and have evolved a complex and multidisciplinary treatment approach, such as surgery, chemotherapy, radiation therapy, targeted therapy, immunotherapy, and so forth (Nagini, 2017; Emens, 2018). However, the prolonged survival trajectory of breast cancer survivors remains complicated and unpredictable by breast cancer recurrence or treatment-related physical effects (Lucas et al., 2017). As a result of these posttreatment late effects, about 30% of breast cancer survivors have reported social difficulties, poorer mental health, physical function decline, and poorer quality of life (Falisi et al., 2017).

Around one third of cancer deaths are caused by the following lifestyle choices: low levels of physical activity, low fruit and vegetable intake, high body mass index (BMI), smoking, and alcohol consumption (Lacombe et al., 2019). Malnutrition is a common finding in cancer patients, and their nutritional status is an important factor influencing their prognosis depending on the clinical type, pathological stage, curative treatment, and the individual patient (Bumrungpert et al., 2018; Clemente et al., 2018). Nutritional immune status is closely related to many aspects of tumors. In Li’s study, it was found that high levels of globulin (GLB) were correlated with poor survival in patients with rectal cancer (Li et al., 2015). The albumin-to-globulin ratio (AGR) was an independent prognostic factor for both overall survival (OS) and cancer-specific survival (CSS) for patients with localized or locally advanced clear cell renal cell carcinoma (CCRCC), and patients with low AGR had poorer OS and CSS (Chen et al., 2017). Moreover, BMI has been proven as an independent prognostic factor for breast cancer, and patients with very high or low BMI have poorer survival compared with normal-weight patients (Bhaskaran et al., 2014; Cespedes Feliciano et al., 2017). Malnutrition and poor immune status may increase the risk of postoperative complications, decrease the response to antitumor therapy, and be associated with poor survival (Liu et al., 2015; Okadome et al., 2020).

Neoadjuvant chemotherapy (NACT) is the standard of care for breast cancer with aggressive biological features (Li et al., 2017). NACT can improve the resectability of locally advanced breast cancer and inflammatory breast cancer, decrease the pathology stage and improve the feasibility and cosmetic effect of breast-conserving surgery, and decrease morbidity and the extent of axillary surgery in women with significant nodal disease (Echeverria et al., 2019). Despite the vast amount of NACT regimens having been conducted in the treatment of breast cancer, there is no internationally generally accepted NACT regimen for patients with advanced breast carcinoma (Wu et al., 2019). Some biomarkers that have been proven are applied to evaluate the treatment efficacy and prognosis of patients with locally advanced breast cancer who are receiving neoadjuvant therapy. In molecular subtypes of breast cancer, the estrogen receptor (ER) status, progesterone receptor (PR) status, Ki-67 status, and human epidermal growth factor receptor-2 (HER-2) status are also critical for the prognosis of breast cancer. Nevertheless, these indicators are usually expensive and time-consuming and achieved from the primary tumor sample (Zheng et al., 2017). Therefore, it is of importance to search easily accessible and reliable markers of breast cancer to evaluate treatment efficacy and provide a better prognosis factor.

Prognostic nutritional index (PNI), which is calculated as serum albumin (ALB) (g/L) + total lymphocyte count (10^9^/L), is initially used to evaluate the nutritional status in patients undergoing surgery (Buzby et al., 1980). The PNI has been reported to be related to the therapeutic effects and predict the survival of various solid tumors (Sun et al., 2014, 2017; Nakatani et al., 2017). In colorectal cancer, a low PNI is an independent poor prognostic factor and is related to poor clinical outcomes (Mohri et al., 2013). In hepatocellular carcinoma, PNI is associated with the prognosis (Pinato et al., 2012). Although a low PNI is found to be related to poor survival in breast cancer, the PNI has been rarely studied in breast cancer patients with NACT treatment. Hence, our study aimed to analyze the prognostic significance of PNI in patients with breast cancer receiving NACT and the relationship between PNI and treatment efficacy.

## Materials and Methods

### Study Population

The retrospective analysis included data from 477 patients with breast cancer who received NACT from January 1998 and December 2016; they form the neoadjuvant chemotherapy group (NACT group). As controls, we also enrolled 308 patients with pathology-proven breast cancer who were diagnosed from January 1998 and December 2016; they form the non-neoadjuvant chemotherapy group (non-NACT group). All enrolled patients were undergoing primary tumor resection at the Cancer Hospital Chinese Academy of Medical Sciences. The clinicopathological features, detailed treatment, and follow-up information were extracted from the medical records of the patients. This study was approved by the ethics committee of the Cancer Hospital Chinese Academy of Medical Sciences. It complied with the standards of the Declaration of Helsinki and its subsequent amendments or similar ethical standards. All patients provided written informed consent before the study.

Patients were included on the basis of the following criteria: (1) with breast cancer based on core needle biopsy before NACT treatment; (2) Karnofsky Performance Score (KPS) ≥ 80 and Performance Status (Zubrod-ECOG-WHO, ZPS) ranging from 0 to 2; (3) had operation after NACT; (4) had complete medical record and follow-up information; (5) survived more than 3 months; and (6) blood samples were obtained within 1 week before NACT treatment.

Patients were excluded on the basis of the following criteria: (1) had received anti-inflammatory medications, such as chemotherapy, radiotherapy, endocrine therapy, targeted therapy, immunotherapy, and so forth; (2) with synchronous and metachronous tumors or distant metastases; (3) with serious complications or any form of acute and chronic inflammatory disease; and (4) who had blood product transfusion within 1 month before NACT treatment.

### Chemotherapy Protocols

Anthracycline-based and/or taxane-based NACT regimens were used for these patients, and every cycle was for 3 weeks: anthracyclines (A) (Zhejiang Hisun Pharmaceutical Co., Ltd., Taizhou, China), cyclophosphamide (C) (Baxter Oncology GmbH, Halle, Germany), 5-fluorouracil (F) (Tianjin Jinyao Pharmaceutical Co., Ltd., China), taxol (T) (Jiangsu Hengrui Medicine Co., Ltd., Lianyungang, China), and platinum compounds (P) (Bristol-Myers Squibb Biopharmaceutical Company, S.r.l., Italy). The following regimens (and doses) were used: AC regimen: 90 mg/m^2^ A and 600 mg/m^2^ C; ACF regimen: 90 mg/m^2^ A, 600 mg/m^2^ C, and 500 mg/m^2^ F; CT regimen: 600 mg/m^2^ C and 175 mg/m^2^ T; ACT regimen: 90 mg/m^2^ A, 600 mg/m^2^ C, and 175 mg/m^2^ A; AT regimen: 90 mg/m^2^ A and 175 mg/m^2^ T; and TP regimen: 175 mg/m^2^ T and AUC 4–6 for P.

### Pretreatment Evaluation, TNM Classification, and Response Evaluation

Pretreatment evaluation included medical history, clinical examination, and routine blood tests. Staging was performed according to the eighth edition of American Joint Committee on Cancer (AJCC) and the Union for International Cancer Control (UICC) TNM stage classification (Fouad et al., 2017; Abdel-Rahman, 2018). Response rates were determined using the Response Evaluation Criteria in Solid Tumors (RECIST) guidelines (Eisenhauer et al., 2009). Histological response was determined with the Miller and Payne grade (MPG) (Del Prete et al., 2019). The toxicity of NACT was evaluated according to the National Cancer Institute Common Toxicity Criteria (NCI-CTC) (Huynh-Le et al., 2014). The lymph vessel invasion and neural invasion of breast cancer were diagnosed by hematoxylin and eosin (HE) staining. Breast cancer molecular subtypes were classified as luminal A, luminal B HER2-positive, luminal B HER2-negative, HER2-enriched, and triple negative (Howlader et al., 2018).

### Peripheral Venous Blood Parameters

Peripheral venous blood samples were collected within 7 days before the first round of NACT. PNI is calculated as serum ALB (g/L) + 5 × total lymphocyte count (10^9^/L). Hematologic parameters were analyzed by an XE-2100 hematology analyzer (Sysmex, Kobe, Japan).

### Follow-Up

All enrolled patients were treated as inpatients and outpatients every 3 months for the first to the second year after operation, every 6 months for the third to the fifth year after operation, then yearly thereafter and until death. Follow-up modalities included clinical examination with laboratory tests (routine blood and blood biochemical tests), ultrasonography of the breast, mammography, and some other examinations, as deemed fit. Disease-free survival (DFS) was defined as the time from the date of surgery to the date of local recurrence or distant metastases, death from any cause, or last follow-up. Overall survival (OS) was defined as the time from the date of surgery to the date of death from any cause or last follow-up.

### Statistical Analysis

The clinicopathologic categorical variables were presented as absolute values and percentages and were compared *via* the chi-square test or Fisher’s exact test. The receiver operating characteristic (ROC) curve was used to determine the optimal cutoff value, and the area under the curve was evaluated by the predictive value. The ratio closest to the point with maximum sensitivity and specificity was defined as the optimal cutoff value. The survival rates, including DFS and OS, were analyzed using Kaplan–Meier plots and compared using the log-rank test. A univariate and multivariate Cox proportional hazards regression model was accessed for the independent prognostic factors, and hazard ratios (HRs) and 95% confidence intervals (CIs) were used to evaluate the association between PNI and breast cancer prognosis. All statistical analyses were performed using the SPSS software (version 17.0, SPSS Inc., Chicago, IL, United States) and GraphPad prism software (version 8.0; GraphPad Inc., La Jolla, CA, United States). Alpha was set at 0.05, and a two-tailed *P* < 0.05 was considered statistically significant.

## Results

### Demographic and Clinicopathologic Characteristics of All Breast Cancer Patients

The clinical and demographic attributes of the patients are shown in Supplementary Table 1. A total of 785 breast cancer patients were enrolled in this study: 477 breast cancer patients were assigned to the NACT group and 308 breast cancer patients were assigned to the non-NACT group. The ROC curve was used to determine the optimal cutoff value of PNI. The optimum cutoff value was 51, and this value was used for all analyses. Then, the patients were stratified into two groups by the optimal cutoff value of PNI: the low PNI group (PNI < 51) and the high PNI group (PNI ≥ 51). All enrolled patients were females. The median age of all breast cancer patients was 47 years, with range from 22 to 82 years. There were 253 breast cancer patients (32.23%) in the low PNI group and 532 breast cancer patients (67.77%) in the high PNI group. Furthermore, there were 167 breast cancer patients (35.01%) with a low PNI and 310 breast cancer patients (64.99%) with a high PNI in the NACT group and 86 breast cancer patients (27.92%) with a low PNI and 222 breast cancer patients (72.08%) with a high PNI in the non-NACT group. With respect to the clinical stage at diagnosis, 92 (11.72%), 382 (48.66%), and 311 (39.62%) breast cancer patients had stage I, II, and III disease, respectively. There were 493 premenopausal breast cancer patients and 292 postmenopausal breast cancer patients. The pathological stage was Tis/T0 in 74 (9.43%) patients, I in 157 (20.00%) patients, II in 262 (33.38%) patients, and III in 292 (37.20%) patients. Of all enrolled patients, statistically significant differences were found between the patients with low PNI and those with high PNI in marital status (χ^2^ = 6.603, *P* = 0.010), post-chemotherapy regimen (χ^2^ = 11.260, *P* = 0.047), type of surgery (χ^2^ = 7.150, *P* = 0.008), pathological T stage (χ^2^ = 13.330, *P* = 0.010), and pathological TNM stage (χ^2^ = 9.303, *P* = 0.026). No statistically significant differences were observed in the clinicopathological characteristics of the other parameters in all enrolled patients (*P* > 0.05). In the NACT group, statistically significant differences were found between the patients with low PNI and those with high PNI in marital status (χ^2^ = 5.739, *P* = 0.017) and MPG (χ^2^ = 14.930, *P* = 0.005). These findings are shown in Supplementary Table 1.

### Nutritional Parameters and Blood Parameters

We chose alanine transaminase (ALT), aspartate transaminase (AST), lactate dehydrogenase (LDH), gamma-glutamyltransferase (GGT), alkaline phosphatase (ALP), glucose (GLU), immunoglobulin A (IgA), IgG, IgM, and ALB as parameters to evaluate the nutritional status of breast cancer patients. The median ALT, AST, LDH, GGT, ALP, GLU, IgA, IgG, IgM, and ALB values were 15.00, 18.00, 167.00, 17.00, and 64.00 U/L, 5.33 mmol/L, and 2.30, 11.70, 1.10, and 45.2 g/L, respectively. We have also chosen C-reactive protein (CRP), cancer antigen (CA)125, CA153, carcinoembryonic antigen (CEA), D-dimer (D-D), fibrinogen (FIB), international normalized ratio (INR), fibrin degradation product (FDP), white blood cell (W), red blood cell (R), hemoglobin (Hb), neutrophil (N), lymphocyte (L), monocyte (M), eosinophils (E), basophils (B), and platelet (P) counts as parameters to evaluate the inflammatory status of breast cancer patients. The median CRP, CA125, CA153, CEA, D-D, FIB, INR, FDP, W, R, Hb, N, L, M, E, B, and P counts were 0.20 mg/dl, 13.35 U/ml, 11.63 U/ml, 1.66 ng/ml, 0.29 mg/L, 2.85 g/L, 0.93, 1.40 μg/ml, 6.01 × 10^9^/L, 4.40 × 10^12^/L, 132 g/L, and 3.68 × 10^9^, 1.76 × 10^9^, 0.35 × 10^9^, 0.06 × 10^9^, 0.02 × 10^9^, and 243 × 10^9^/L, respectively. All of these peripheral venous blood parameters were collected before treatment. In the NACT group, there were significant differences in ALB (χ^2^ = 184.400, *P* < 0.0001), CRP (χ^2^ = 9.251, *P* = 0.002), W (χ^2^ = 25.540, *P* < 0.0001), R (χ^2^ = 19.040, *P* < 0.0001), Hb (χ^2^ = 21.100, *P* < 0.0001), N (χ^2^ = 184.400, *P* = 0.008), L (χ^2^ = 17.430, *P* < 0.0001), B (χ^2^ = 8.100, *P* = 0.004), and P (χ^2^ = 8.975, *P* = 0.003). In the non-NACT group, there were significant differences in GGT (χ^2^ = 8.544, *P* = 0.004), IgM (χ^2^ = 5.171, *P* = 0.023), ALB (χ^2^ = 62.690, *P* < 0.0001), CRP (χ^2^ = 4.472, *P* = 0.035), W (χ^2^ = 6.609, *P* = 0.010), R (χ^2^ = 7.808, *P* = 0.005), Hb (χ^2^ = 15.030, *P* = 0.0001), and M (χ^2^ = 6.248, *P* = 0.012). No other significant correlation was found. The correlations between the nutritional parameters/blood parameters and PNI are shown in Supplementary Table 2.

### Univariate and Multivariate Cox Regression Survival Analyses

In univariate analysis, ALB, CA153, lymphocyte, PNI, type of surgery, histologic grade, pathological T stage, pathological N stage, pathological TNM stage, molecular subtype, HER2 status, Ki-67 status, CK5/6 status, TOP2A status, lymph vessel invasion, postoperative endocrine therapy, and postoperative targeted therapy were the significant prognostic factors for DFS and OS. In multivariate Cox regression analysis, ALB, CA153, lymphocyte, PNI, type of surgery, histologic grade, pathological T stage, pathological N stage, pathological TNM stage, molecular subtype, HER2 status, Ki-67 status, TOP2A status, lymph vessel invasion, postoperative endocrine therapy, and postoperative targeted therapy were the significant prognostic factors for DFS and OS. These results are shown in Supplementary Table 3.

### DFS and OS by PNI

**FIGURE 1:**
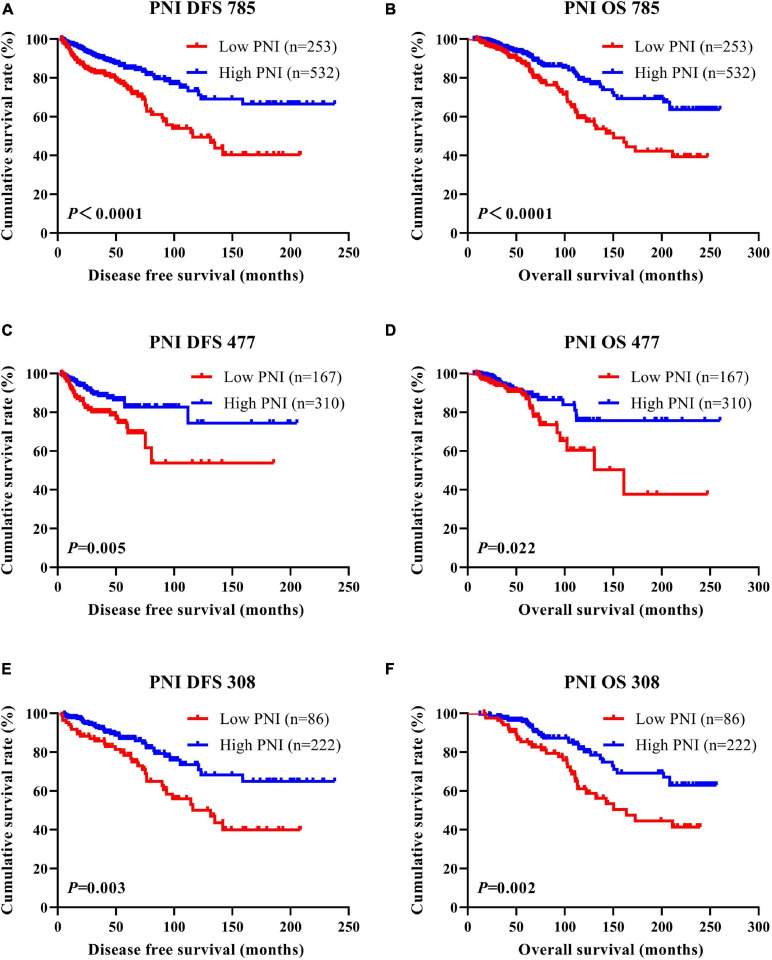
Disease-free survival (DFS) and overall survival (OS) of patients with breast cancer. **(A,B)** Kaplan–Meier analyses of DFS **(A)** and OS **(B)** for the prognostic nutritional index (PNI) of all patients with breast cancer. **(C,D)** Kaplan–Meier analyses of DFS **(C)** and OS **(D)** for the PNI of patients with breast cancer in the neoadjuvant chemotherapy (NACT) group. **(E,F)** Kaplan–Meier analyses of DFS **(E)** and OS **(F)** for the PNI of patients with breast cancer in the non-NACT group.

According to the univariate and multivariate Cox regression analyses, the results indicated that PNI had prognostic significance for DFS and OS using the cutoff value of 51. In univariate analysis, a high PNI was associated with prolonged DFS and OS (*P* < 0.0001, HR = 0.310, 95%CI = 0.194–0.494 and *P* < 0.0001, HR = 0.366, 95%CI = 0.243–0.550, respectively). In multivariate Cox regression analysis, a high PNI was associated with prolonged DFS and OS (*P* < 0.0001, HR = 0.264, 95%CI = 0.160–0.435 and *P* < 0.0001, HR = 0.319, 95%CI = 0.207–0.491, respectively). Of all enrolled breast patients, the mean DFS and OS for patients with low PNI were 36.60 months (range = 3.47–208.57 months) and 64.97 months (range = 9.13–247.33 months), and the mean DFS and OS for patients with high PNI were 47.64 months (range = 3.10–238.00 months) and 73.61 months (range = 6.43–260.03 months), respectively. Furthermore, the mean DFS and OS times for patients with high PNI were longer than for those with low PNI by the log-rank test (χ^2^ = 18.540, *P* < 0.0001 and χ^2^ = 16.060, *P* < 0.0001, respectively; Figures 1A,B). In the NACT group, the mean DFS and OS for patients with low PNI were 44.34 months (range = 3.47–185.63 months) and 65.27 months (range = 9.13–247.33 months), and the mean DFS and OS for patients with high PNI were 49.70 months (range = 3.10–205.47 months) and 79.85 months (range = 6.43–260.03 months), respectively. By the log-rank test, the mean DFS and OS times for patients with high PNI were longer than for those with low PNI (χ^2^ = 8.044, *P* = 0.005 and χ^2^ = 5.285, *P* = 0.022, respectively; Figures 1C,D). In the non-NACT group, the mean DFS and OS for patients with low PNI were 33.83 months (range = 3.53–208.57 months) and 59.75 months (range = 16.90–239.53 months), and the mean DFS and OS for patients with high PNI were 37.00 months (range = 5.73–238.00 months) and 71.20 months (range = 12.57–256.37 months), respectively. By the log-rank test, the mean DFS and OS times for patients with high PNI were longer than for those with low PNI (χ^2^ = 8.960, *P* = 0.003 and χ^2^ = 9.672, *P* = 0.002, respectively; Figures 1E,F).

### Association of Pathologic Stage and PNI in Patients With Breast Cancer

**FIGURE 2:**
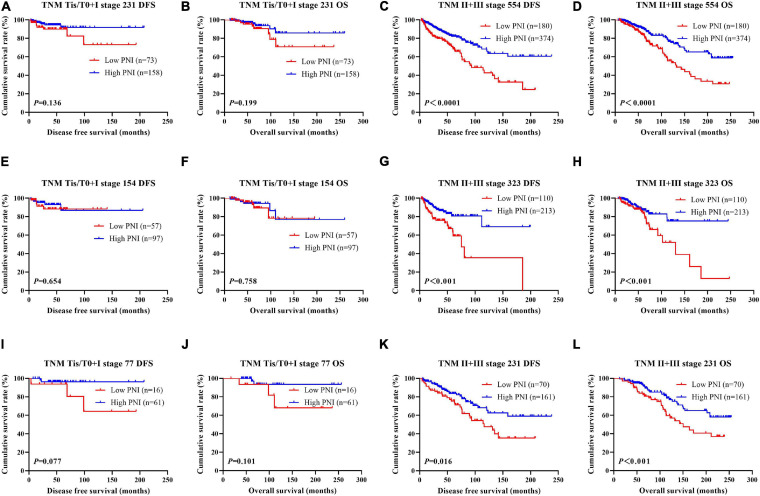
Disease-free survival (DFS) and overall survival (OS) for the prognostic nutritional index (PNI) of patients with breast cancer in different pathologic stages. **(A,B)** Kaplan–Meier analysis of DFS **(A)** and OS **(B)** for the PNI of patients with early stage breast cancer (Tis/T0 + I stage) in all enrolled patients. **(C,D)** Kaplan–Meier analysis of DFS **(C)** and OS **(D)** for the PNI of patients with advanced stage breast cancer (II + III stage) in all enrolled patients. **(E,F)** Kaplan–Meier analysis of DFS **(E)** and OS **(F)** for the PNI of patients with early stage breast cancer (Tis/T0 + I stage) in the neoadjuvant chemotherapy (NACT) group. **(G,H)** Kaplan–Meier analysis of DFS **(G)** and OS **(H)** for the PNI of patients with advanced stage breast cancer (II + III stage) in the NACT group. **(I,J)** Kaplan–Meier analysis of DFS **(I)** and OS **(J)** for the PNI of patients with early stage breast cancer (Tis/T0 + I stage) in the non-NACT group. **(K,L)** Kaplan–Meier analysis of DFS **(K)** and OS **(L)** for the PNI of patients with advanced stage breast cancer (II + III stage) in the non-NACT group.

According to the univariate and multivariate analyses, we found that pathologic T stage, pathologic N stage, and pathologic TNM stage were the significant prognostic factors (Supplementary Table 3). In order to further investigate the prognostic efficiency of PNI, the PNI was analyzed by the pathologic TNM stage. We defined the patients with pathologic Tis/T0 + I stage as early stage breast cancer and the patients with pathologic II + III stage as advanced stage breast cancer, and we used the log-rank test to analyze the PNI with the different pathologic stages. Of all enrolled breast patients, the results indicated that patients with high PNI had longer DFS and OS than those with low PNI in early stage breast cancer (χ^2^ = 2.223, *P* = 0.136 and χ^2^ = 1.650, *P* = 0.199, respectively; Figures 2A,B). Meanwhile, patients with high PNI had longer DFS and OS than those with low PNI in advanced stage breast cancer (χ^2^ = 17.820, *P* < 0.0001 and χ^2^ = 15.390, *P* < 0.0001, respectively; Figures 2C,D). In the NACT group, the results indicated that patients with high PNI had longer DFS and OS than those with low PNI in early stage breast cancer (χ^2^ = 0.201, *P* = 0.654 and χ^2^ = 0.095, *P* = 0.758, respectively; Figures 2E,F). Meanwhile, patients with high PNI had longer DFS and OS than those with low PNI in advanced stage breast cancer (χ^2^ = 11.790, *P* < 0.001 and χ^2^ = 7.119, *P* < 0.001, respectively; Figures 2G,H). In the non-NACT group, the results indicated that patients with high PNI had longer DFS and OS than those with low PNI in early stage breast cancer (χ^2^ = 3.126, *P* = 0.077 and χ^2^ = 2.697, *P* = 0.101, respectively; Figures 2I,J). Meanwhile, patients with high PNI had longer DFS and OS than those with low PNI in advanced stage breast cancer (χ^2^ = 5.801, *P* = 0.016 and χ^2^ = 7.078, *P* = 0.008, respectively; Figures 2K,L).

### Association of Molecular Subtype and PNI in Patients With Breast Cancer

**FIGURE 3:**
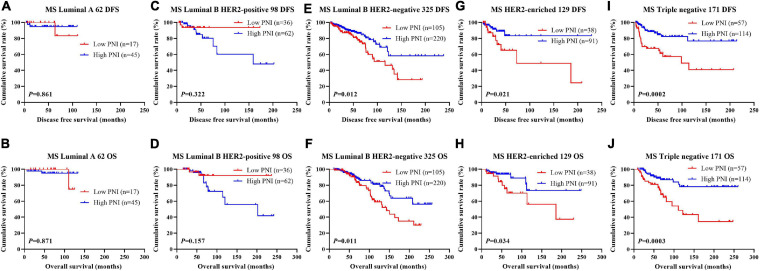
Disease-free survival (DFS) and overall survival (OS) for the prognostic nutritional index (PNI) of patients with breast cancer in different molecular subtypes. **(A,B)** Kaplan–Meier analysis of DFS **(A)** and OS **(B)** for the PNI of patients with luminal A breast cancer in all enrolled patients. **(C,D)** Kaplan–Meier analysis of DFS **(C)** and OS **(D)** for the PNI of patients with luminal B HER2-positive breast cancer in all enrolled patients. **(E,F)** Kaplan–Meier analysis of DFS **(E)** and OS **(F)** for the PNI of patients with luminal B HER2-negative breast cancer in all enrolled patients. **(G,H)** Kaplan–Meier analysis of DFS **(G)** and OS **(H)** for the PNI of patients with HER2-enriched breast cancer in all enrolled patients. **(I,J)** Kaplan–Meier analysis of DFS **(I)** and OS **(J)** for the PNI of patients with triple-negative breast cancer in all enrolled patients.

**FIGURE 4:**
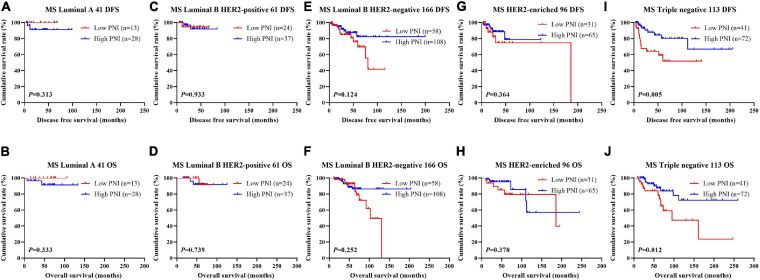
Disease-free survival (DFS) and overall survival (OS) for the prognostic nutritional index (PNI) of patients with breast cancer in different molecular subtypes in the neoadjuvant chemotherapy (NACT) group. **(A,B)** Kaplan-Meier analysis of DFS **(A)** and OS **(B)** for the PNI of patients with luminal A breast cancer in NACT group. **(C,D)** Kaplan-Meier analysis of DFS **(C)** and OS **(D)** for the PNI of patients with luminal B HER2-positive breast cancer in NACT group. **(E,F)** Kaplan-Meier analysis of DFS **(E)** and OS **(F)** for the PNI of patients with luminal B HER2-negative breast cancer in NACT group. **(G,H)** Kaplan-Meier analysis of DFS **(G)** and OS **(H)** for the PNI of patients with HER2-enriched breast cancer in NACT group. **(I,J)** Kaplan-Meier analysis of DFS **(I)** and OS **(J)** for the PNI of patients with triple-negative breast cancer in NACT group.

**FIGURE 5:**
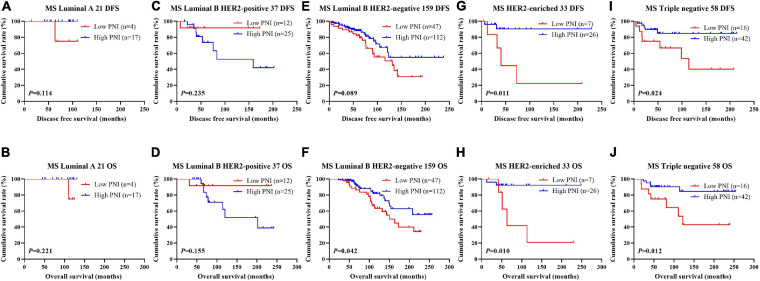
Disease-free survival (DFS) and overall survival (OS) for the prognostic nutritional index (PNI) of patients with breast cancer in different molecular subtypes in the non-neoadjuvant chemotherapy (NACT) group. **(A,B)** Kaplan-Meier analysis of DFS **(A)** and OS **(B)** for the PNI of patients with luminal A breast cancer in non-NACT group. **(C,D)** Kaplan-Meier analysis of DFS **(C)** and OS **(D)** for the PNI of patients with luminal B HER2-positive breast cancer in non-NACT group. **(E,F)** Kaplan-Meier analysis of DFS **(E)** and OS **(F)** for the PNI of patients with luminal B HER2-negative breast cancer in non-NACT group. **(G,H)** Kaplan-Meier analysis of DFS **(G)** and OS **(H)** for the PNI of patients with HER2-enriched breast cancer in non-NACT group. **(I,J)** Kaplan-Meier analysis of DFS **(I)** and OS **(J)** for the PNI of patients with triple-negative breast cancer in non-NACT group.

The results indicated that molecular subtype was the significant prognostic factor by univariate and multivariate analyses (Supplementary Table 3). Of all enrolled patients, 62 cases were luminal A subtype, 98 cases were luminal B HER2-positive subtype, 325 cases were luminal B HER2-negative subtype, 129 cases were HER2-enriched subtype, and 171 cases were triple-negative subtype (Table 1). In order to further evaluate the prognostic value of PNI, the PNI was accessed by the molecular subtypes. The PNI with different molecular subtypes was analyzed by the log-rank test. The mean DFS and OS times for patients with luminal A subtype were 47.31 and 69.72 months, respectively. Moreover, the mean DFS and OS times for patients with high PNI by the log-rank test were longer than for those with low PNI in luminal A subtype (χ^2^ = 0.031, *P* = 0.861 and χ^2^ = 0.026, *P* = 0.871, respectively; Figures 3A,B). The mean DFS and OS times for patients with luminal B HER2-positive subtype were 44.46 and 69.32 months, respectively. Moreover, the mean DFS and OS times for patients with high PNI by the log-rank test were longer than for those with low PNI in luminal B HER2-positive subtype (χ^2^ = 0.979, *P* = 0.322 and χ^2^ = 2.002, *P* = 0.157, respectively; Figures 3C,D). The mean DFS and OS times for patients with luminal B HER2-negative subtype were 42.13 and 63.13 months, respectively. Moreover, the mean DFS and OS times for patients with high PNI by the log-rank test were longer than for those with low PNI in luminal B HER2-negative subtype (χ^2^ = 6.268, *P* = 0.012 and χ^2^ = 6.457, *P* = 0.011, respectively; Figures 3E,F). The mean DFS and OS times for patients with HER2-enriched subtype were 24.30 and 49.40 months, respectively. Moreover, the mean DFS and OS times for patients with high PNI by the log-rank test were longer than for those with low PNI in HER2-enriched subtype (χ^2^ = 5.291, *P* = 0.021 and χ^2^ = 4.488, *P* = 0.034, respectively; Figures 3G,H). The mean DFS and OS times for patients with triple-negative subtype were 37.07 and 60.73 months, respectively. Moreover, the mean DFS and OS times for patients with high PNI by the log-rank test were longer than for those with low PNI in triple-negative subtype (χ^2^ = 13.690, *P* < 0.001 and χ^2^ = 12.980, *P* < 0.001, respectively; Figures 3I,J). The DFS and OS for the PNI of breast cancer patients with different molecular subtypes in the NACT and non-NACT groups are shown in Figures 4, 5.

**TABLE 1:** Association of molecular subtypes and prognostic nutritional index (PNI) in patients with breast cancer.

Parameters	PNI (*N* = 785)					PNI (*n* = 477)					PNI (*n* = 308)				

	*N*	Low PNI (*n* = 253)	High PNI (*n* = 532)	χ^2^	*P* value	*N*	Low PNI (*n* = 167)	High PNI (*n* = 310)	χ^2^	*P* value	*N*	Low PNI (*n* = 86)	High PNI (*n* = 222)	χ^2^	*P* value
Core needle biopsy (*N* = 477)															
Molecular subtypes									2.454	0.653					
Luminal A						25 (5.24%)	12 (7.19%)	13 (4.19%)							
Luminal B HER2+						67 (14.05%)	22 (13.17%)	45 (14.52%)							
Luminal B HER2−						186 (38.99%)	62 (37.13%)	124 (40.00%)							
HER2 enriched						91 (19.08%)	31 (18.56%)	60 (19.35%)							
Triple negative						108 (22.64%)	40 (23.95%)	68 (21.94%)							
ER status									0.049	0.825					
Negative						191 (40.04%)	68 (40.72%)	123 (39.68%)							
Positive						286 (59.96%)	99 (59.28%)	187 (60.32%)							
ER status									1.179	0.758					
0–25%						228 (47.80%)	82 (49.10%)	146 (47.10%)							
26–50%						42 (8.80%)	17 (10.18%)	25 (8.06%)							
51–75%						33 (6.92%)	10 (5.99%)	23 (7.42%)							
76–100%						174 (36.48%)	58 (34.73%)	116 (37.42%)							
PR status									0.309	0.579					
Negative						189 (39.62%)	69 (41.32%)	120 (38.71%)							
Positive						288 (60.38%)	98 (58.68%)	190 (61.29%)							
PR status									0.082	0.994					
0–25%						286 (59.96%)	100 (59.88%)	186 (60.00%)							
26–50%						67 (14.05%)	24 (14.37%)	43 (13.87%)							
51–75%						45 (9.43%)	15 (8.98%)	30 (9.68%)							
76–100%						79 (16.56%)	28 (16.77%)	51 (16.45%)							
HER2 status									0.007	0.934					
Negative (0– + + )						313 (65.62%)	110 (65.87%)	203 (65.48%)							
Positive ( + + + )						164 (34.38%)	57 (34.13%)	107 (34.52%)							
Ki-67 status									0.426	0.514					
Negative (≤14%)						84 (17.61%)	32 (19.16%)	52 (16.77%)							
Positive (>14%)						393 (82.39%)	135 (80.84%)	258 (83.23%)							
Ki-67 status									1.477	0.688					
0–25%						161 (33.75%)	55 (32.93%)	106 (34.19%)							
26–50%						189 (39.62%)	66 (39.52%)	123 (39.68%)							
51–75%						88 (18.45%)	29 (17.37%)	59 (19.03%)							
76–100%						39 (8.18%)	17 (10.18%)	22 (7.10%)							
Postoperative pathology (IHC)															
Molecular subtype				2.118	0.714				1.093	0.895				2.149	0.708
Luminal A	62 (7.90%)	17 (6.72%)	45 (8.46%)			41 (8.60%)	13 (7.78%)	28 (9.02%)			21 (6.82%)	4 (4.65%)	17 (7.66%)		
Luminal B HER2+	98 (12.48%)	36 (14.23%)	62 (11.65%)			61 (12.79%)	24 (14.37%)	37 (11.94%)			37 (12.01%)	12 (13.95%)	25 (11.26%)		
Luminal B HER2-	325 (41.40%)	105 (41.50%)	220 (41.35%)			166 (34.80%)	58 (34.74%)	108 (34.84%)			159 (51.63%)	47 (54.66%)	112 (50.45%)		
HER2 enriched	129 (16.44%)	38 (15.02%)	91 (17.11%)			96 (20.12%)	31 (18.56%)	65 (20.97%)			33 (10.71%)	7 (8.14%)	26 (11.71%)		
Triple negative	171 (21.78%)	57 (22.53%)	114 (21.43%)			113 (23.69%)	41 (24.55%)	72 (23.23%)			58 (18.83%)	16 (18.60%)	42 (18.92%)		
ER status				0.049	0.826				0.003	0.958				0.355	0.552
Negative	296 (37.71%)	94 (37.15%)	202 (37.97%)			195 (40.88%)	68 (40.72%)	127 (40.97%)			101 (32.79%)	26 (30.23%)	75 (33.78%)		
Positive	489 (62.29%)	159 (62.85%)	330 (62.03%)			282 (59.12%)	99 (59.28%)	183 (59.03%)			207 (67.21%)	60 (69.77%)	147 (66.22%)		
ER status				3.722	0.293				0.841	0.840				5.508	0.138
0–25%	375 (47.77%)	129 (50.99%)	246 (46.24%)			235 (49.27%)	85 (50.90%)	150 (48.39%)			140 (45.46%)	44 (51.16%)	96 (43.24%)		
26–50%	66 (8.41%)	25 (9.88%)	41 (7.71%)			31 (6.50%)	12 (7.19%)	19 (6.13%)			35 (11.36%)	13 (15.12%)	22 (9.91%)		
51–75%	48 (6.11%)	13 (5.14%)	35 (6.58%)			27 (5.66%)	10 (5.99%)	17 (5.48%)			21 (6.82%)	3 (3.49%)	18 (8.11%)		
76–100%	296 (37.71%)	86 (33.99%)	210 (39.47%)			184 (38.57%)	60 (35.92%)	124 (40.00%)			112 (36.36%)	26 (30.23%)	86 (38.74%)		
PR status				0.154	0.694				0.009	0.926				1.339	0.247
Negative	315 (40.13%)	99 (39.13%)	216 (40.60%)			210 (44.03%)	74 (44.31%)	136 (43.87%)			105 (34.09%)	25 (29.07%)	80 (36.04%)		
Positive	470 (59.87%)	154 (60.87%)	316 (59.40%)			267 (55.97%)	93 (55.69%)	174 (56.13%)			203 (65.91%)	61 (70.93%)	142 (63.96%)		
PR status				0.546	0.909				0.426	0.935				1.558	0.669
0–25%	502 (63.95%)	161 (63.64%)	341 (64.10%)			335 (70.23%)	116 (69.46%)	219 (70.65%)			167 (54.22%)	45 (52.33%)	122 (54.95%)		
26–50%	90 (11.46%)	31 (12.25%)	59 (11.09%)			48 (10.06%)	16 (9.58%)	32 (10.32%)			42 (13.64%)	15 (17.44%)	27 (12.16%)		
51–75%	55 (7.01%)	19 (7.51%)	36 (6.76%)			38 (7.97%)	15 (8.98%)	23 (7.42%)			17 (5.52%)	4 (4.65%)	13 (5.86%)		
76–100%	138 (17.58%)	42 (16.60%)	96 (18.05%)			56 (11.74%)	20 (11.98%)	36 (11.61%)			82 (26.62%)	22 (25.58%)	60 (27.03%)		
HER2 status				0.065	0.799				0.045	0.833				0.062	0.804
Negative (0– + + )	557 (70.96%)	178 (70.36%)	379 (71.24%)			320 (67.09%)	111 (66.47%)	209 (67.42%)			237 (76.95%)	67 (77.91%)	170 (76.58%)		
Positive ( + + + )	228 (29.04%)	75 (29.64%)	153 (28.76%)			157 (32.91%)	56 (33.53%)	101 (32.58%)			71 (23.05%)	19 (22.09%)	52 (23.42%)		
Ki-67 status				0.566	0.452				0.538	0.463				4.138	0.042
Negative (≤14%)	219 (27.90%)	75 (29.64%)	144 (27.07%)			153 (32.08%)	50 (29.94%)	103 (33.23%)			66 (21.43%)	25 (29.07%)	41 (18.47%)		
Positive (>14%)	566 (72.10%)	178 (70.36%)	388 (72.93%)			324 (67.92%)	117 (70.06%)	207 (66.77%)			242 (78.57%)	61 (70.93%)	181 (81.53%)		
Ki-67 status				2.780	0.427				2.920	0.404				0.689	0.876
0–25%	342 (43.57%)	114 (45.06%)	228 (42.86%)			233 (48.84%)	82 (49.10%)	151 (48.71%)			109 (35.39%)	32 (37.21%)	77 (34.68%)		
26–50%	257 (32.74%)	74 (29.25%)	183 (34.40%)			139 (29.14%)	43 (25.75%)	96 (30.97%)			118 (38.31%)	31 (36.05%)	87 (39.19%)		
51–75%	137 (17.45%)	50 (19.76%)	87 (16.35%)			70 (14.68%)	30 (17.96%)	40 (12.90%)			67 (21.75%)	20 (23.25%)	47 (21.17%)		
76–100%	49 (6.24%)	15 (5.93%)	34 (6.39%)			35 (7.34%)	12 (7.19%)	23 (7.42%)			14 (4.55%)	3 (3.49%)	11 (4.96%)		
AR status				6.920	0.009				11.730	<0.001				0.017	0.896
Negative	666 (84.84%)	227 (89.72%)	439 (82.52%)			362 (75.89%)	142 (85.03%)	220 (70.97%)			304 (98.70%)	85 (98.84%)	219 (98.65%)		
Positive	119 (15.16%)	26 (10.28%)	93 (17.48%)			115 (24.11%)	25 (14.97%)	90 (29.03%)			4 (1.30%)	1 (1.16%)	3 (1.35%)		
AR status				6.354	0.096				10.260	0.017				0.044	0.834
0–25%	688 (87.65%)	232 (91.70%)	456 (85.71%)			383 (80.29%)	147 (88.02%)	236 (76.13%)			305 (99.03%)	85 (98.84%)	220 (99.10%)		
26–50%	25 (3.18%)	4 (1.58%)	21 (3.95%)			25 (5.24%)	4 (2.40%)	21 (6.77%)			0 (0.00%)	0 (0.00%)	0 (0.00%)		
51–75%	29 (3.69%)	6 (2.37%)	23 (4.32%)			29 (6.08%)	6 (3.59%)	23 (7.42%)			0 (0.00%)	0 (0.00%)	0 (0.00%)		
76–100%	43 (5.48%)	11 (4.35%)	32 (6.02%)			40 (8.39%)	10 (5.99%)	30 (9.68%)			3 (0.97%)	1 (1.16%)	2 (0.90%)		
CK5/6 status				0.109	0.741				1.248	0.264				2.092	0.148
Negative	684 (87.13%)	219 (86.56%)	465 (87.41%)			406 (85.12%)	138 (82.63%)	268 (86.45%)			278 (90.26%)	81 (94.19%)	197 (88.74%)		
Positive	101 (12.87%)	34 (13.44%)	67 (12.59%)			71 (14.88%)	29 (17.37%)	42 (13.55%)			30 (9.74%)	5 (5.81%)	25 (11.26%)		
E-cad status				8.716	0.003				3.612	0.057				11.140	<0.001
Negative	353 (44.97%)	133 (52.57%)	220 (41.35%)			170 (35.64%)	69 (41.32%)	101 (32.58%)			183 (59.42%)	64 (74.42%)	119 (53.60%)		
Positive	432 (55.03%)	120 (47.43%)	312 (58.65%)			307 (64.36%)	98 (58.68%)	209 (67.42%)			125 (40.58%)	22 (25.58%)	103 (46.40%)		
EGFR status				2.078	0.150				0.130	0.719				7.281	0.007
Negative	589 (75.03%)	198 (78.26%)	391 (73.50%)			335 (70.23%)	119 (71.26%)	216 (69.68%)			254 (82.47%)	79 (91.86%)	175 (78.83%)		
Positive	196 (24.97%)	55 (21.74%)	141 (26.50%)			142 (29.77%)	48 (28.74%)	94 (30.32%)			54 (17.53%)	7 (8.14%)	47 (21.17%)		
P53 status				1.381	0.240				0.137	0.712				1.994	0.158
Negative	395 (50.32%)	135 (53.36%)	260 (48.87%)			243 (50.94%)	87 (52.10%)	156 (50.32%)			152 (49.35%)	48 (55.81%)	104 (46.85%)		
Positive	390 (49.68%)	118 (46.64%)	272 (51.13%)			234 (49.06%)	80 (47.90%)	154 (49.68%)			156 (50.65%)	38 (44.19%)	118 (53.15%)		
P53 status				1.575	0.665				4.173	0.243				0.634	0.729
0–25%	576 (73.38%)	183 (72.33%)	393 (73.87%)			353 (74.00%)	118 (70.66%)	235 (75.81%)			223 (72.41%)	65 (75.58%)	158 (71.17%)		
26–50%	80 (10.19%)	23 (9.09%)	57 (10.72%)			45 (9.44%)	14 (8.38%)	31 (10.00%)			35 (11.36%)	9 (10.47%)	26 (11.71%)		
51–75%	108 (13.75%)	39 (15.42%)	69 (12.97%)			58 (12.16%)	27 (16.17%)	31 (10.00%)			50 (16.23%)	12 (13.95%)	38 (17.12%)		
76–100%	21 (2.68%)	8 (3.16%)	13 (2.44%)			21 (4.40%)	8 (4.79%)	13 (4.19%)			0 (0.00%)	0 (0.00%)	0 (0.00%)		
TOP2A status				4.598	0.032				0.024	0.877				15.940	<0.0001
Negative	299 (38.09%)	110 (43.48%)	189 (35.53%)			165 (34.59%)	57 (34.13%)	108 (34.84%)			134 (43.51%)	53 (61.63%)	81 (36.49%)		
Positive	486 (61.91%)	143 (56.52%)	343 (64.47%)			312 (65.41%)	110 (65.87%)	202 (65.16%)			174 (56.49%)	33 (38.37%)	141 (63.51%)		
TOP2A status				0.408	0.939				1.974	0.578				3.368	0.338
0–25%	575 (73.25%)	187 (73.91%)	388 (72.92%)			354 (74.21%)	120 (71.86%)	234 (75.48%)			221 (71.76%)	67 (77.90%)	154 (69.37%)		
26–50%	158 (20.13%)	48 (18.97%)	110 (20.68%)			88 (18.45%)	31 (18.56%)	57 (18.39%)			70 (22.73%)	17 (19.77%)	53 (23.87%)		
51–75%	49 (6.24%)	17 (6.72%)	32 (6.02%)			33 (6.92%)	15 (8.98%)	18 (5.81%)			16 (5.19%)	2 (2.33%)	14 (6.31%)		
76–100%	3 (0.38%)	1 (0.40%)	2 (0.38%)			2 (0.42%)	1 (0.60%)	1 (0.32%)			1 (0.32%)	0 (0.00%)	1 (0.45%)		
Lymph vessel invasion				0.756	0.385				0.001	0.995				3.936	0.047
Negative	558 (71.08%)	185 (73.12%)	373 (70.11%)			320 (67.09%)	112 (67.07%)	208 (67.10%)			238 (77.27%)	73 (84.88%)	165 (74.32%)		
Positive	227 (28.92%)	68 (26.88%)	159 (29.89%)			157 (32.91%)	55 (32.93%)	102 (32.90%)			70 (22.73%)	13 (15.12%)	57 (25.68%)		
Neural invasion				0.041	0.840				0.018	0.892				0.005	0.944
Negative	670 (85.35%)	215 (84.98%)	455 (85.53%)			384 (80.50%)	135 (80.84%)	249 (80.32%)			286 (92.86%)	80 (93.02%)	206 (92.79%)		
Positive	115 (14.65%)	38 (15.02%)	77 (14.47%)			93 (19.50%)	32 (19.16%)	61 (19.68%)			22 (7.14%)	6 (6.98%)	16 (7.21%)		

**ER*, estrogen receptor; *PR*, progesterone receptor; *AR*, androgen receptor; *EFGR*, epidermal growth factor receptor; *IHC*, immunohistochemistry.*

### Association of Lymph Vessel Invasion (LVI) and PNI in Patients With Breast Cancer

**FIGURE 6:**
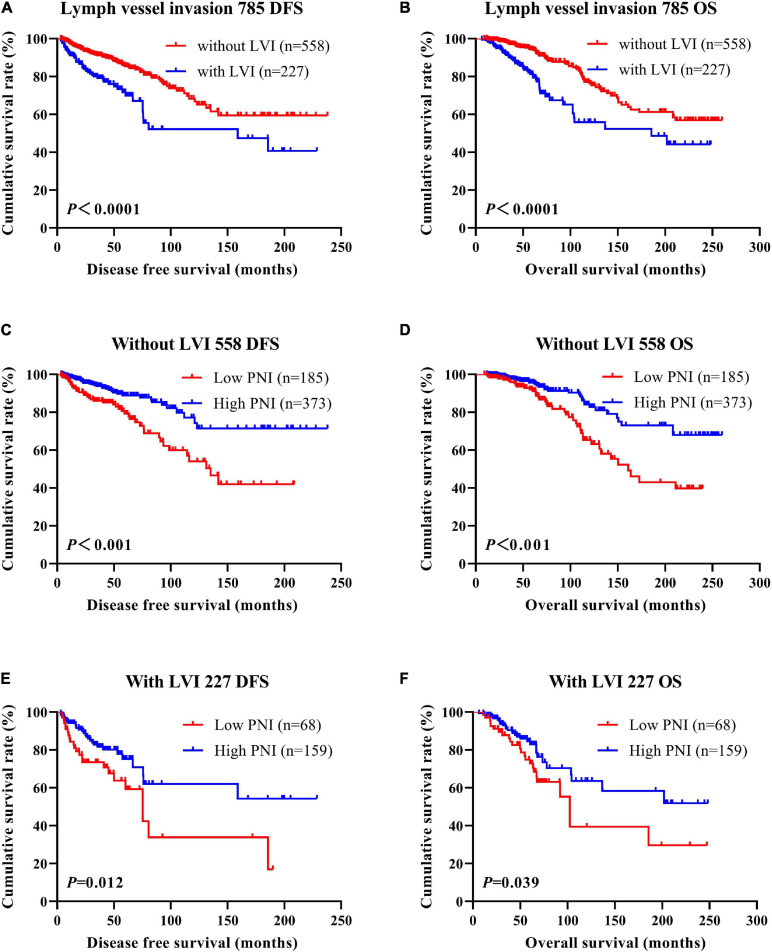
Disease-free survival (DFS) and overall survival (OS) of patients with breast cancer by lymph vessel invasion status. **(A,B)** Kaplan–Meier analysis of the DFS **(A)** and OS **(B)** of breast cancer patients by lymph vessel invasion status. **(C,D)** Kaplan–Meier analysis of the DFS **(C)** and OS **(D)** of patients without lymph vessel invasion by prognostic nutritional index (PNI). **(E,F)** Kaplan–Meier analysis of the DFS **(E)** and OS **(F)** of patients with lymph vessel invasion by PNI.

**FIGURE 7:**
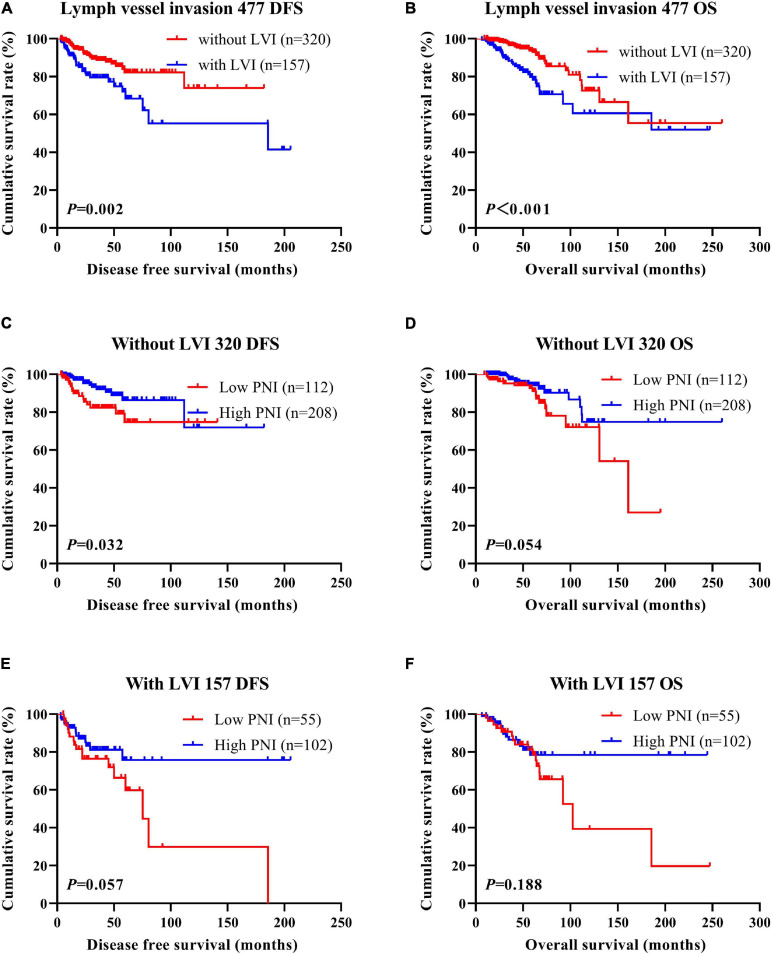
Disease-free survival (DFS) and overall survival (OS) of patients with breast cancer by lymph vessel invasion status in the neoadjuvant chemotherapy (NACT) group. **(A,B)** Kaplan-Meier analysis of the DFS **(A)** and OS **(B)** of breast cancer patients by lymph vessel invasion status in NACT group. **(C,D)** Kaplan-Meier analysis of the DFS **(C)** and OS **(D)** of patients without lymph vessel invasion by prognostic nutritional index (PNI) in NACT group. **(E,F)** Kaplan-Meier analysis of the DFS **(E)** and OS **(F)** of patients with lymph vessel invasion by PNI in NACT group.

**FIGURE 8:**
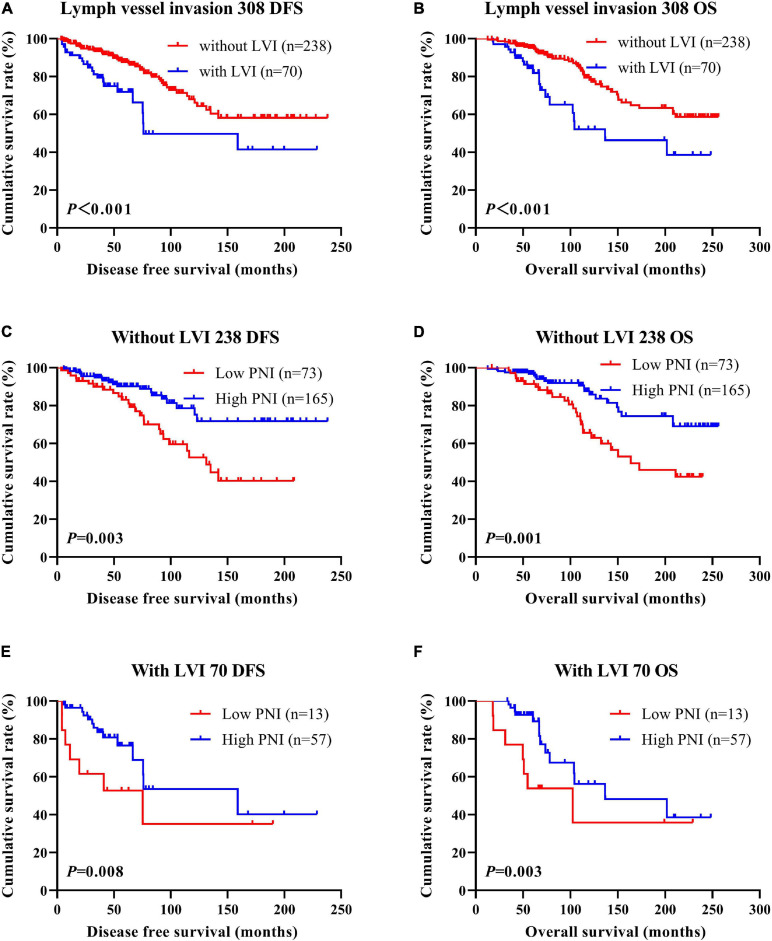
Disease-free survival (DFS) and overall survival (OS) of patients with breast cancer by lymph vessel invasion status in the non-neoadjuvant chemotherapy (NACT) group. **(A,B)** Kaplan-Meier analysis of the DFS **(A)** and OS **(B)** of breast cancer patients by lymph vessel invasion status in non-NACT group. **(C,D)** Kaplan-Meier analysis of the DFS **(C)** and OS **(D)** of patients without lymph vessel invasion by prognostic nutritional index (PNI) in non-NACT group. **(E,F)** Kaplan-Meier analysis of the DFS **(E)** and OS **(F)** of patients with lymph vessel invasion by PNI in non-NACT group.

According to the univariate and multivariate analyses, lymph vessel invasion was a significant prognostic factor (Supplementary Table 3). For the sake of further studying the prognostic efficiency of PNI, we analyzed the lymph vessel invasion by PNI. The lymph vessel invasion status was divided into without lymph vessel invasion and with lymph vessel invasion. The mean DFS and OS times of all enrolled patients without lymph vessel invasion were 50.96 and 79.65 months, respectively, and those with lymph vessel invasion were 28.97 and 53.37 months, respectively. The mean DFS and OS times of patients without lymph vessel invasion were longer than of those patients with lymph vessel invasion by the log-rank test (χ^2^ = 20.940, *P* < 0.0001 and χ^2^ = 26.540, *P* < 0.0001, respectively; Figures 6A,B). The mean DFS and OS times of patients without lymph vessel invasion were 41.47 and 66.60 months with low PNI and 64.75 and 108.00 months with high PNI, respectively. The results indicated that the mean DFS and OS times of patients with high PNI by the log-rank test were longer than of those patients with low PNI without lymph vessel invasion (χ^2^ = 14.520, *P* < 0.001 and χ^2^ = 14.120, *P* < 0.001, respectively; Figures 6C,D). The mean DFS and OS times of patients with lymph vessel invasion were 26.75 and 34.82 months with low PNI and 29.50 and 61.15 months with high PNI, respectively. The results indicated that the mean DFS and OS times of patients with high PNI by the log-rank test were longer than of those patients with low PNI with lymph vessel invasion (χ^2^ = 6.266, *P* = 0.012 and χ^2^ = 4.270, *P* = 0.039, respectively; Figures 6E,F). The DFS and OS for the PNI of breast cancer patients without or with lymph vessel invasion in the NACT and non-NACT groups are shown in Figures 7, 8.

### Association of PNI and Neoadjuvant Chemotherapy or Postoperative Chemotherapy

In the NACT group, 28 patients received the AC/ACF regimen, 27 patients received the CT/ACT regimen, 223 patients received the AT regimen, 141 patients received the TP regimen, and 58 patients received other regimens. After operation, there were 230 patients undergoing postoperative chemotherapy, and 247 patients did not receive postoperative chemotherapy. Forty-three patients received the AC/ACF regimen, 30 patients received the CT/ACT regimen, 37 patients received the AT regimen, 39 patients received the TP regimen, and 81 patients received other regimens. The clinical objective response rate [complete response (CR) + partial response (PR)] was 66.88% (319/477) and the clinical benefit rate [CR + PR + stable disease (SD)] was 98.53% (470/477); the non-clinical response rate [SD + partial disease (PD)] was 33.12% (158/477). We also used the MPG system to evaluate the pathological response. The grade 1 rate was 4.61% (22/477), the grade 2 rate was 26.42% (126/477), the grade 3 rate was 37.11% (177/477), the grade 4 rate was 13.00% (62/477), and the grade 5 rate was 18.87% (90/477). The pathologic complete response (pCR) rate was 15.09% (72/477) and the non-pCR rate was 84.91% (405/477).

**FIGURE 9:**
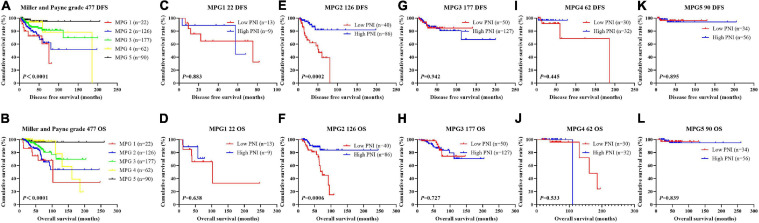
Disease-free survival (DFS) and overall survival (OS) for the prognostic nutritional index (PNI) of patients with breast cancer in Miller and Payne grade (MPG) in the neoadjuvant chemotherapy (NACT) group. **(A,B)** Kaplan-Meier analysis of DFS **(A)** and OS **(B)** for the PNI of patients with different MPG in NACT group. **(C,D)** Kaplan-Meier analysis of DFS **(C)** and OS **(D)** for the PNI of patients with MPG 1 in NACT group. **(E,F)** Kaplan-Meier analysis of DFS **(E)** and OS **(F)** for the PNI of patients with MPG 2 in NACT group. **(G,H)** Kaplan-Meier analysis of DFS **(G)** and OS **(H)** for the PNI of patients with MPG 3 in NACT group. **(I,J)** Kaplan-Meier analysis of DFS **(I)** and OS **(J)** for the PNI of patients with MPG 4 in NACT group. **(K,L)** Kaplan-Meier analysis of DFS **(I)** and OS **(J)** for the PNI of patients with MPG 5 in NACT group.

**FIGURE 10:**
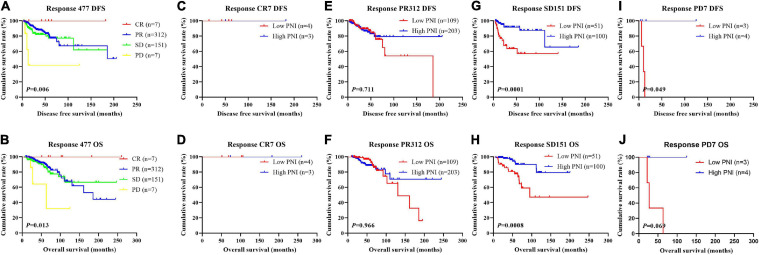
Disease-free survival (DFS) and overall survival (OS) for the prognostic nutritional index (PNI) of patients with breast cancer in response status in the neoadjuvant chemotherapy (NACT) group. **(A,B)** Kaplan-Meier analysis of DFS **(A)** and OS **(B)** for the PNI of patients with different Response in NACT group. **(C,D)** Kaplan-Meier analysis of DFS **(C)** and OS **(D)** for the PNI of patients with Response CR in NACT group. **(E,F)** Kaplan-Meier analysis of DFS **(E)** and OS **(F)** for the PNI of patients with Response PR in NACT group. **(G,H)** Kaplan-Meier analysis of DFS **(G)** and OS **(H)** for the PNI of patients with Response SD in NACT group. **(I,J)** Kaplan-Meier analysis of DFS **(I)** and OS **(J)** for the PNI of patients with Response PD in NACT group.

In order to further evaluate the prognostic efficiency of PNI, we analyzed the PNI by MPG. The PNI with different MPG grades was analyzed by the log-rank test. There were significant differences in the different MPG grades on the mean DFS and OS times by using the log-rank test (χ^2^ = 18.290, *P* < 0.0001 and χ^2^ = 18.020, *P* < 0.0001, respectively). Moreover, the results indicated that the mean DFS and OS times of patients with high PNI were longer than of those with low PNI in the MPG 2 group (χ^2^ = 13.980, *P* = 0.0002 and χ^2^ = 11.800, *P* = 0.0006, respectively; Figure 9). We also analyzed the response by PNI, and there were significant differences in the response on the mean DFS and OS times by using the log-rank test (χ^2^ = 12.540, *P* = 0.006 and χ^2^ = 10.820, *P* = 0.013, respectively). Furthermore, the results indicated that the mean DFS and OS times of patients with high PNI were longer than of those with low PNI in the SD response status (χ^2^ = 14.390, *P* = 0.0001 and χ^2^ = 11.250, *P* = 0.0008, respectively; Figure 10).

### Correlation Between PNI and Toxicity Assessment

In the NACT group, we evaluated and analyzed the toxicities after receiving NACT for two cycles. The common toxicities included decreased appetite, nausea, vomiting, diarrhea, mouth ulcers, alopecia, peripheral neurotoxicity, anemia, leukopenia, neutropenia, thrombocytopenia, gastrointestinal reaction, myelosuppression, and hepatic dysfunction (Table 2). There were no chemotherapy-related deaths in this study. The results indicated that there were significant differences using the PNI cutoff value of 51 in anemia (χ^2^ = 7.064, *P* = 0.029), leukopenia (χ^2^ = 13.570, *P* = 0.001), and myelosuppression (χ^2^ = 7.066, *P* = 0.029).

**TABLE 2:** Correlations between PNI and toxicity assessment.

Parameters	PNI (*n* = 477)				
	*N*	Low PNI (*n* = 167)	High PNI (*n* = 310)	χ^2^	*P* value
Decreased appetite				0.898	0.343
No	70 (14.68%)	28 (16.77%)	42 (13.55%)		
Yes	407 (85.32%)	139 (83.23%)	268 (86.45%)		
Nausea				1.136	0.286
No	59 (12.37%)	17 (10.18%)	42 (13.55%)		
Yes	418 (87.63%)	150 (89.82%)	268 (86.45%)		
Vomiting				2.315	0.128
No	234 (49.06%)	74 (44.31%)	160 (51.61%)		
Yes	243 (50.94%)	93 (55.69%)	150 (48.39%)		
Diarrhea				0.299	0.584
No	444 (93.08%)	154 (92.22%)	290 (93.55%)		
Yes	33 (6.92%)	13 (7.78%)	20 (6.45%)		
Mouth ulcers				1.424	0.233
No	463 (97.07%)	160 (95.81%)	303 (97.74%)		
Yes	14 (2.93%)	7 (4.19%)	7 (2.26%)		
Alopecia				0.003	0.958
No	222 (46.54%)	78 (46.71%)	144 (46.45%)		
Yes	255 (53.46%)	89 (53.29%)	166 (53.55%)		
Peripheral neurotoxicity				0.132	0.717
No	390 (81.76%)	138 (82.63%)	252 (81.29%)		
Yes	87 (18.24%)	29 (17.37%)	58 (18.71%)		
Anemia				7.064	0.029
Grade 0	257 (53.88%)	77 (46.10%)	180 (58.06%)		
Grades 1–2	215 (45.07%)	87 (52.10%)	128 (41.29%)		
Grades 3–4	5 (1.05%)	3 (1.80%)	2 (0.65%)		
Leukopenia				13.570	0.001
Grade 0	138 (28.93%)	31 (18.56%)	107 (34.52%)		
Grades 1–2	233 (48.85%)	95 (56.89%)	138 (44.52%)		
Grades 3–4	106 (22.22%)	41 (24.55%)	65 (20.96%)		
Neutropenia				5.679	0.059
Grade 0	143 (29.98%)	39 (23.35%)	104 (33.55%)		
Grades 1–2	179 (37.53%)	71 (42.51%)	108 (34.84%)		
Grades 3–4	155 (32.49%)	57 (34.13%)	98 (31.61%)		
Thrombocytopenia				0.511	0.774
Grade 0	372 (77.99%)	128 (76.65%)	244 (78.71%)		
Grades 1–2	98 (20.55%)	37 (22.15%)	61 (19.68%)		
Grades 3–4	7 (1.46%)	2 (1.20%)	5 (1.61%)		
Gastrointestinal reaction				2.347	0.309
Grade 0	38 (7.97%)	9 (5.39%)	29 (9.35%)		
Grades 1–2	433 (90.78%)	156 (93.41%)	277 (89.35%)		
Grades 3–4	6 (1.25%)	2 (1.20%)	4 (1.30%)		
Myelosuppression				7.066	0.029
Grade 0	90 (18.87%)	21 (12.57%)	69 (22.26%)		
Grades 1–2	175 (36.69%)	63 (37.72%)	112 (36.13%)		
Grades 3–4	212 (44.44%)	83 (49.71%)	129 (41.61%)		
Hepatic dysfunction				0.612	0.736
Grade 0	371 (77.78%)	129 (77.25%)	242 (78.07%)		
Grades 1–2	105 (22.01%)	38 (22.75%)	67 (21.61%)		
Grades 3–4	1 (2.10%)	0 (0.00%)	1 (0.32%)		

## Discussion

Breast cancer is the most commonly diagnosed cancer among women all over the world and is a major public health problem worldwide (Siegel et al., 2020). Despite newer therapies in the recent years, recurrence and metastasis remain the main challenges of cancer management. In China, as a result of late diagnosis, about 30–40% of invasive breast cancer patients will eventually develop into metastatic breast cancer, and patients have a low 5-year survival rate of less than 30% (Early Breast Cancer Trialists’ Collaborative Group et al., 2012; Noguchi et al., 2020). Although there were recent improvements in early detection and progress in surgical techniques and multimodal therapy, and the clinical outcomes and quality of life have improved, breast cancer remains the leading cause of cancer death for women (Waks and Winer, 2019). Moreover, some breast cancer patients still develop recurrence and metastasis even after curative resection and neoadjuvant/adjuvant therapy (Tabor et al., 2020). Therefore, accurate prediction of prognosis is needed to improve patient survival and identify those patients who are more likely to benefit from neoadjuvant chemotherapy.

In this study, the clinical and demographic attributes of the 785 breast cancer patients enrolled were analyzed. The results indicated that a high PNI was significantly associated with marital status, post-chemotherapy regimen, type of surgery, pathological T stage, pathological TNM stage, AR status, E-cad status, and the TOP2A status of all enrolled patients. We also found that a high PNI was significantly associated with marital status and MPG in the NACT group and with tumor site, United States primary tumor site, operative time, type of surgery, tumor size, and pathological T stage in the non-NACT group. Moreover, we also analyzed the nutritional parameters and blood parameters. The results indicated that a high PNI was significantly associated with IgG, ALB, W, R, Hb, N, L, M, B, and P of all enrolled patients. Moreover, a high PNI was significantly associated with ALB, CRP, W, R, Hb, N, L, B, and P in the NACT group and with GGT, IgM, ALB, CRP, W, R, Hb, and M in the non-NACT group.

Several studies have investigated the prognostic value of the PNI in breast cancer patients (Yang et al., 2014; Wang et al., 2019; Hua et al., 2020). However, these studies did not determine the PNI among patients who had received neoadjuvant therapy. In the present study, we evaluated the prognostic impact of the PNI in breast cancer patients who received neoadjuvant chemotherapy followed by operation. The preoperative PNI was an independent prognostic factor of DFS and OS by univariate and multivariate Cox regression survival analyses. The results also indicated that the mean DFS and OS times of patients with high PNI were longer than of those with low PNI by the log-rank test in the NACT and non-NACT groups. The mechanism by which a low nutritional status decreases the survival time of breast cancer patients is still not sufficiently understood. The PNI, as a systemic immune–nutrition index based on the peripheral lymphocyte count and serum albumin levels, represents the immune and nutritional status and is also a significant biomarker for many tumors (Dai et al., 2020; Hao et al., 2020; Zhang et al., 2020). The PNI was initially developed to evaluate the postoperative complications in patients who received gastrointestinal surgery (Onodera et al., 1984). Lymphocytes and serum albumin are significantly closely related to the prognosis of cancer patients (Otagiri et al., 2020; Powell et al., 2020). Lymphocytes play an active role in the adaptive immune system to clear tumors from the body and to prevent their development and spread (Hanahan and Weinberg, 2011). Serum albumin has been reported to reflect an individual’s nutrition and inflammatory status (Fuhrman et al., 2004; Yoshida et al., 2020). It is generally known that low levels of serum albumin and lymphocytes promote inflammatory tumor development and the spread and metastasis of cancer (Saroha et al., 2013; Mendez et al., 2016).

We also analyzed the association of pathologic stage and PNI in patients with breast cancer and observed that those with high PNI had longer DFS and OS than those with low PNI in early stage and advanced stage breast cancer in all enrolled breast cancer patients. Furthermore, patients with high PNI had longer DFS and OS than those with low PNI in early stage and advanced stage breast cancer, especially in those with advanced stage breast cancer in the NACT and non-NACT groups. Apart from these analyses, we also observed that patients with high PNI had longer DFS and OS than those with low PNI in different molecular subtypes, except for luminal B HER2-positive subtype. Lymph vessel invasion (LVI) was the significant prognostic factor by univariate and multivariate analyses. The results indicated that patients without lymph vessel invasion survive longer than those patients with lymph vessel invasion, and the mean DFS and OS times of patients with high PNI were longer than of those patients with low PNI with lymph vessel invasion status. Moreover, patients with lymph vessel invasion and low PNI value had worse survival times. In Sahoo’s study, the results indicated that poor overall as well as disease-free survival and overall survival pattern were observed for LVI-positive patients as compared with LVI-negative patients; LVI and PNI constitute potential targets for the treatment of breast cancer patients (Sahoo et al., 2014). In He’s study, it was indicated that LVI was an independent poor prognostic factor for the development of recurrence in lymph node-negative breast cancer and was also promising in determining prognosis and treatment strategies (He et al., 2017). Another study indicated that both positive lymphatic invasion (LI) and positive vascular invasion (VI) showed inferior OS and DFS compared with negative LI and negative VI; moreover, both positive LI and positive VI showed worse survival rates in the luminal A and triple-negative subtypes (Hwang et al., 2017).

In the NACT group, all breast cancer patients could tolerate the neoadjuvant chemotherapy toxicities, and the regimens were safe and effective. The common toxicities after neoadjuvant chemotherapy were hematologic and gastrointestinal reaction, and the results indicated that, in toxicity assessment, there was no difference in these toxicities using the cutoff value of 51 for PNI, except in anemia, leukopenia, and myelosuppression.

However, there are several limitations that cannot be neglected in this study. Firstly, this is a retrospective design and was conducted in a single center with a limited number of patients. A multicenter study and more patients should be enrolled. Secondly, selection bias cannot be excluded, even if consecutive patients are included and eligibility criteria are implemented to minimize the bias. Thirdly, the PNI is a nonspecific tumor marker because other non-cancer diseases can be confused with a tumor. Further validation in a large prospective study is needed to further assess the prognostic and predictive value of PNI for patients with breast cancer in the future.

## Conclusion

Prognostic nutritional index is a significant prognostic factor for patients with breast cancer and can effectively predict the survival and prognosis of breast cancer. It is of importance to take into consideration the unbalanced distribution of medical conditions in China, and noninvasive, reproducible, and convenient biomarkers should be used for the prevention and treatment of breast cancer.

## Data Availability Statement

The raw data supporting the conclusions of this article will be made available by the authors, without undue reservation.

## Ethics Statement

The studies involving human participants were reviewed and approved by This study was approved by the Ethics Committee of Cancer Hospital Chinese Academy of Medical Sciences. The patients/participants provided their written informed consent to participate in this study.

## Author Contributions

LC and PB wrote the original draft and did the review and editing. XK and SH did the formal analysis. ZW did the data curation. XW contributed to the investigation. YF contributed to the methodology and supervision. JW helped with the resources, funding acquisition, and project administration. All authors contributed to the article and approved the submitted version.

## Conflict of Interest

The authors declare that the research was conducted in the absence of any commercial or financial relationships that could be construed as a potential conflict of interest.
