# Supplementary material for: Prognostic Nutritional Index (PNI) in Patients With Breast Cancer Treated With Neoadjuvant Chemotherapy as a Useful Prognostic Indicator
Source: Front Cell Dev Biol. 2021 Mar 30;9:656741. doi: 10.3389/fcell.2021.656741 (PMC8042235; doi:10.3389/fcell.2021.656741)
Supplement: Supplementary file 1 [file Table_1.pdf]

Supplementary Table 1 Demographic and clinicopathologic characteristics of 785 patients with breast cancer

Parameters	N	PNI 785				N	PNI 477				N	PNI 308			
Cases (n)	785	Low PNI 253	High PNI 532	χ2	P value		Low PNI 167	High PNI 310	χ2	P value		Low PNI 86	High PNI 222	χ2	P value
Age (years)				1.649	0.199				3.347	0.067				0.134	0.714
＜47	386(49.17%)	116(45.85%)	270(50.75%)			230(48.22%)	71(42.51%)	159(51.29%)			156(50.65%)	45(52.33%)	111(50.00%)		
⩾47	399(50.83%)	137(54.15%)	262(49.25%)			247(51.78%)	96(57.49%)	151(48.71%)			152(49.35%)	41(47.67%)	111(50.00%)		
Marital status				6.603	0.010				5.739	0.017				1.302	0.254
Married	756(96.31%)	250(98.81%)	506(95.11%)			457(95.81%)	165(98.80%)	292(94.19%)			299(97.08%)	85(98.84%)	214(96.40%)		
Unmarried	29(3.69%)	3(1.19%)	26(4.89%)			20(4.19%)	2(1.20%)	18(5.81%)			9(2.92%)	1(1.16%)	8(3.60%)		
Occupation				4.906	0.086				1.548	0.461				4.360	0.113
Mental worker	358(45.61%)	122(48.22%)	236(44.36%)			238(49.90%)	84(50.30%)	154(49.68%)			120(38.96%)	38(44.19%)	82(36.93%)		
Manual worker	125(15.92%)	47(18.58%)	78(14.66%)			66(13.84%)	27(16.17%)	39(12.58%)			59(19.16%)	20(23.25%)	39(17.57%)		
Others	302(38.47%)	84(33.20%)	218(40.98%)			173(36.26%)	56(33.53%)	117(37.74%)			129(41.88%)	28(32.56%)	101(45.50%)		
Weight (Kg)				0.005	0.947				0.191	0.662				0.181	0.670
＜62.00	383(48.79%)	123(48.62%)	260(48.87%)			235(49.27%)	80(47.90%)	155(50.00%)			148(48.05%)	43(50.00%)	105(47.30%)		
⩾62.00	402(51.21%)	130(51.38%)	272(51.13%)			242(50.73%)	87(52.10%)	155(50.00%)			160(51.95%)	43(50.00%)	117(52.70%)		
Height (M)				0.273	0.601				0.812	0.368				0.338	0.561
＜1.60	337(42.93%)	112(44.27%)	225(42.29%)			218(45.70%)	81(48.50%)	137(44.19%)			119(38.64%)	31(36.05%)	88(39.64%)		
⩾1.60	448(57.07%)	141(55.73%)	307(57.71%)			259(54.30%)	86(51.50%)	173(55.81%)			189(61.36%)	55(63.95%)	134(60.36%)		
BMI				0.005	0.941				0.841	0.359				1.160	0.282
＜24.00	391(49.81%)	126(49.80%)	265(49.81%)			245(51.36%)	81(48.50%)	164(52.90%)			146(47.40%)	45(52.33%)	101(45.50%)		
⩾24.00	394(50.19%)	127(50.20%)	267(50.19%)			232(48.64%)	86(51.50%)	146(47.10%)			162(52.60%)	41(47.67%)	121(54.50%)		
Family history				0.675	0.411				0.107	0.744				1.857	0.173
No	597(76.05%)	197(77.87%)	400(75.19%)			347(72.75%)	123(73.65%)	224(72.26%)			250(81.17%)	74(86.05%)	176(79.28%)		
Yes	188(23.95%)	56(22.13%)	132(24.81%)			130(27.25%)	44(26.35%)	86(27.74%)			58(18.83%)	12(13.95%)	46(20.72%)		
Menarche age				1.672	0.196				2.829	0.093				0.005	0.943
＜14	308(39.24%)	91(35.97%)	217(40.79%)			196(41.09%)	60(35.93%)	136(43.87%)			112(36.36%)	31(36.05%)	81(36.49%)		

⩾14	477(60.76%)	162(64.03%)	315(59.21%)			281(58.91%)	107(64.07%)	174(56.13%)			196(63.64%)	55(63.95%)	141(63.51%)		
Menopause				0.597	0.440				1.382	0.240				0.483	0.487
No	493(62.80%)	154(60.87%)	339(63.72%)			280(58.70%)	92(55.09%)	188(60.65%)			213(69.16%)	62(72.09%)	151(68.02%)		
Yes	292(37.20%)	99(39.13%)	193(36.28%)			197(41.30%)	75(44.91%)	122(39.35%)			95(30.84%)	24(27.91%)	71(31.98%)		
ABO blood type				1.043	0.791				0.889	0.828				1.615	0.656
A	214(27.26%)	67(26.48%)	147(27.63%)			132(27.67%)	42(25.14%)	90(29.03%)			82(26.62%)	25(29.07%)	57(25.68%)		
B	262(33.38%)	80(31.62%)	182(34.21%)			145(30.40%)	52(31.14%)	93(30.00%)			117(37.99%)	28(32.56%)	89(40.08%)		
O	234(29.81%)	80(31.62%)	154(28.95%)			146(30.61%)	54(32.34%)	92(29.68%)			88(28.57%)	26(30.23%)	62(27.93%)		
AB	75(9.55%)	26(10.28%)	49(9.21%)			54(11.32%)	19(11.38%)	35(11.29%)			21(6.82%)	7(8.14%)	14(6.31%)		
Tumor site				1.928	0.165				0.007	0.935				4.214	0.040
Right	369(47.01%)	128(50.59%)	241(45.30%)			233(48.85%)	82(49.10%)	151(48.71%)			136(44.16%)	46(53.49%)	90(40.54%)		
Left	416(52.99%)	125(49.41%)	291(54.70%)			244(51.15%)	85(50.90%)	159(51.29%)			172(55.84%)	40(46.51%)	132(59.46%)		
US-Primary tumor site				3.199	0.525				2.136	0.711				12.640	0.013
Upper outer quadrant	489(62.29%)	158(62.45%)	331(62.22%)			305(63.94%)	109(65.27%)	196(63.23%)			184(59.74%)	49(56.98%)	135(60.81%)		
Lower outer quadrant	81(10.32%)	26(10.28%)	55(10.34%)			44(9.22%)	18(10.78%)	26(8.39%)			37(12.01%)	8(9.30%)	29(13.06%)		
Lower inner quadrant	37(4.71%)	10(3.95%)	27(5.08%)			18(3.77%)	6(3.59%)	12(3.87%)			19(6.17%)	4(4.65%)	15(6.76%)		
Upper inner quadrant	120(15.29%)	35(13.83%)	85(15.97%)			61(12.80%)	17(10.18%)	44(14.19%)			59(19.16%)	18(20.93%)	41(18.47%)		
Central	58(7.39%)	24(9.49%)	34(6.39%)			49(10.27%)	17(10.18%)	32(10.32%)			9(2.92%)	7(8.14%)	2(0.90%)		
US-Tumor size				3.980	0.137				0.386	0.825				2.900	0.235
⩽2cm	310(39.49%)	88(34.78%)	222(41.73%)			135(28.30%)	45(26.95%)	90(29.03%)			175(56.82%)	43(50.00%)	132(59.46%)		
＞2 and ＜5cm	402(51.21%)	137(54.15%)	265(49.81%)			273(57.23%)	96(57.49%)	177(57.10%)			129(41.88%)	41(47.67%)	88(39.64%)		
⩾5cm	73(9.30%)	28(11.07%)	45(8.46%)			69(14.47%)	26(15.57%)	43(13.87%)			4(1.30%)	2(2.33%)	2(0.90%)		
US-LNM				0.058	0.809				0.678	0.410				0.033	0.857
No	579(73.76%)	188(74.31%)	391(73.50%)			314(65.83%)	114(68.26%)	200(64.52%)			265(86.04%)	74(86.05%)	191(86.04%)		
Yes	206(26.24%)	65(25.69%)	141(26.50%)			163(34.17%)	53(31.74%)	110(35.48%)			43(13.96%)	12(13.95%)	31(13.96%)		
US-BIRADS				2.304	0.680				1.533	0.821				1.974	0.578
4a	7(0.89%)	1(0.40%)	6(1.13%)			7(1.47%)	1(0.60%)	6(1.94%)			0(0.00%)	0(0.00%)	0(0.00%)		
4b	17(2.17%)	6(2.37%)	11(2.06%)			13(2.73%)	5(2.99%)	8(2.58%)			4(1.30%)	1(1.16%)	3(1.35%)		
4c	54(6.88%)	14(5.53%)	40(7.52%)			34(7.13%)	11(6.59%)	23(7.42%)			20(6.49%)	3(3.49%)	17(7.66%)		

5	320(40.76%)	103(40.71%)	217(40.79%)			183(38.36%)	65(38.92%)	118(38.06%)			137(44.48%)	38(44.19%)	99(44.59%)		
6	387 (49.30%)	129(50.99%)	258(48.50%)			240(50.31%)	85(50.90%)	155(50.00%)			147(47.73%)	44(51.16%)	103(46.40%)		
Clinical T stage				0.670	0.880				2.001	0.572				2.941	0.401
T1	181(23.06%)	55(21.74%)	126(23.68%)			67(14.05%)	28(16.77%)	39(12.57%)			114(37.01%)	27(31.40%)	87(39.19%)		
T2	401(51.08%)	132(52.17%)	269(50.56%)			225(47.17%)	79(47.31%)	146(47.10%)			176(57.14%)	53(61.62%)	123(55.41%)		
T3	130(16.56%)	44(17.39%)	86(16.17%)			114(23.90%)	38(22.75%)	76(24.52%)			16(5.19%)	6(6.98%)	10(4.50%)		
T4	73(9.30%)	22(8.70%)	51(9.59%)			71(14.88%)	22(13.17%)	49(15.81%)			2(0.65%)	0(0.00%)	2(0.90%)		
Clinical N stage				1.899	0.594				1.130	0.770				1.101	0.777
N0	300(38.22%)	89(35.18%)	211(39.66%)			73(15.30%)	22(13.17%)	51(16.45%)			227(73.70%)	67(77.91%)	160(72.07%)		
N1	232(29.55%)	76(30.04%)	156(29.32%)			164(34.38%)	60(35.93%)	104(33.55%)			68(22.08%)	16(18.60%)	52(23.42%)		
N2	160(20.38%)	54(21.34%)	106(19.92%)			151(31.66%)	52(31.14%)	99(31.94%)			9(2.92%)	2(2.33%)	7(3.16%)		
N3	93(11.85%)	34(13.44%)	59(11.09%)			89(18.66%)	33(19.76%)	56(18.06%)			4(1.30%)	1(1.16%)	3(1.35%)		
Clinical TNM stage				2.531	0.282				0.473	0.789				1.397	0.498
I	92(11.72%)	24(9.49%)	68(12.78%)			14(2.94%)	6(3.59%)	8(2.58%)			78(25.32%)	18(20.93%)	60(27.03%)		
II	382(48.66%)	121(47.83%)	261(49.06%)			168(35.22%)	57(34.13%)	111(35.81%)			214(69.48%)	64(74.42%)	150(67.57%)		
III	311(39.62%)	108(42.68%)	203(38.26%)			295(61.84%)	104(62.28%)	191(61.61%)			16(5.20%)	4(4.65%)	12(5.40%)		
Neoadjuvant Chemotherapy															
Chemotherapy regimen									5.568	0.234					
AC/ACF						28(5.87%)	7(4.19%)	21(6.77%)							
CT/ACT						27(5.66%)	9(5.39%)	18(5.81%)							
AT						223(46.75%)	87(52.10%)	136(43.87%)							
TP						141(29.56%)	41(24.55%)	100(32.26%)							
Others						58(12.16%)	23(13.77%)	35(11.29%)							
Chemotherapy times									3.765	0.052					
＜6						134(28.09%)	56(33.53%)	78(25.16%)							
⩾6						343(71.91%)	111(66.47%)	232(74.84%)							
Response									1.799	0.615					
CR						7(1.47%)	4(2.40%)	3(0.97%)							
PR						312(65.41%)	109(65.26%)	203(65.48%)							

SD						151(31.65%)	51(30.54%)	100(32.26%)							
PD						7(1.47%)	3(1.80%)	4(1.29%)							
Miller and Payne grade									14.930	0.005					
1						22(4.61%)	13(7.78%)	9(2.90%)							
2						126(26.42%)	40(23.95%)	86(27.74%)							
3						177(37.11%)	50(29.95%)	127(40.98%)							
4						62(13.00%)	30(17.96%)	32(10.32%)							
5						90(18.86%)	34(20.36%)	56(18.06%)							
Pathological response									0.231	0.631					
pCR						72(15.09%)	27(16.17%)	45(14.52%)							
non-pCR						405(84.91%)	140(83.83%)	265(85.48%)							
Post-chemotherapy regimen				11.260	0.047				8.430	0.134				6.471	0.263
AC/ACF	125(15.92%)	37(14.62%)	88(16.54%)			43(9.01%)	10(5.99%)	33(10.65%)			82(26.62%)	27(31.40%)	55(24.77%)		
CT/ACT	125(15.92%)	30(11.86%)	95(17.86%)			30(6.29%)	7(4.19%)	23(7.42%)			95(30.84%)	23(26.74%)	72(32.43%)		
AT	97(12.36%)	36(14.23%)	61(11.47%)			37(7.76%)	17(10.18%)	20(6.45%)			60(19.48%)	19(22.09%)	41(18.47%)		
TP	61(7.77%)	14(5.53%)	47(8.83%)			39(8.18%)	11(6.59%)	28(9.03%)			22(7.14%)	3(3.49%)	19(8.56%)		
Others	108(13.76%)	43(17.00%)	65(12.22%)			81(16.98%)	33(19.76%)	48(15.48%)			27(8.78%)	10(11.63%)	17(7.66%)		
NO	269(34.27%)	93(36.76%)	176(33.08%)			247(51.78%)	89(53.29%)	158(50.97%)			22(7.14%)	4(4.65%)	18(8.11%)		
Operative time (min)				2.776	0.096				1.600	0.206				3.995	0.046
＜90	347(44.20%)	101(39.92%)	246(46.24%)			253(53.04%)	82(49.10%)	171(55.16%)			94(30.52%)	19(22.09%)	75(33.78%)		
⩾90	438(55.80%)	152(60.08%)	286(53.76%)			224(46.96%)	85(50.90%)	139(44.84%)			214(69.48%)	67(77.91%)	147(66.22%)		
Type of surgery				7.150	0.008				0.594	0.441				5.939	0.015
Mastectomy	606(77.20%)	210(83.00%)	396(74.44%)			406(85.12%)	145(86.83%)	261(84.19%)			200(64.94%)	65(75.58%)	135(60.81%)		
Breast-conserving surgery	179(22.80%)	43(17.00%)	136(25.56%)			71(14.88%)	22(13.17%)	49(15.81%)			108(35.06%)	21(24.42%)	87(39.19%)		
Tumor size (cm)				5.975	0.050				1.401	0.496				10.880	0.004
⩽2cm	437(55.67%)	131(51.78%)	306(57.52%)			263(55.14%)	92(55.09%)	171(55.16%)			174(56.50%)	39(45.35%)	135(60.81%)		
＞2 and ＜5cm	299(38.09%)	99(39.13%)	200(37.59%)			172(36.06%)	57(34.13%)	115(37.10%)			127(41.23%)	42(48.84%)	85(38.29%)		
⩾5cm	49(6.24%)	23(9.09%)	26(4.89%)			42(8.80%)	18(10.78%)	24(7.74%)			7(2.27%)	5(5.81%)	2(0.90%)		
Histologic type				0.982	0.612				2.473	0.290				0.464	0.793

Ductal	758(96.56%)	245(96.83%)	513(96.43%)			461(96.64%)	163(97.60%)	298(96.13%)			297(96.43%)	82(95.35%)	215(96.85%)		
Lobular	13(1.66%)	5(1.98%)	8(1.50%)			7(1.47%)	3(1.80%)	4(1.29%)			6(1.95%)	2(2.33%)	4(1.80%)		
Others	14(1.78%)	3(1.19%)	11(2.07%)			9(1.89%)	1(0.60%)	8(2.58%)			5(1.62%)	2(2.33%)	3(1.35%)		
Histologic grade				0.197	0.906				1.592	0.451				5.040	0.081
I	133(16.94%)	45(17.78%)	88(16.54%)			108(22.64%)	34(20.36%)	74(23.87%)			25(8.12%)	11(12.79%)	14(6.31%)		
II	431(54.90%)	138(54.55%)	293(55.08%)			244(51.15%)	84(50.30%)	160(51.61%)			187(60.71%)	54(62.79%)	133(59.91%)		
III	221(28.15%)	70(27.67%)	151(28.38%)			125(26.21%)	49(29.34%)	76(24.52%)			96(31.17%)	21(24.42%)	75(33.78%)		
Pathological TNM classification															
Pathological T stage				13.330	0.010				7.809	0.099				11.350	0.023
Tis/T0	92(11.72%)	34(13.44%)	58(10.90%)			88(18.45%)	33(19.76%)	55(17.74%)			4(1.30%)	1(1.16%)	3(1.35%)		
T1	302(38.47%)	92(36.36%)	210(39.47%)			190(39.83%)	69(41.32%)	121(39.03%)			112(36.36%)	23(26.74%)	89(40.09%)		
T2	326(41.53%)	94(37.15%)	232(43.61%)			149(31.24%)	41(24.55%)	108(34.84%)			177(57.47%)	53(61.63%)	124(55.86%)		
T3	45(5.73%)	23(9.10%)	22(4.14%)			34(7.13%)	16(9.58%)	18(5.81%)			11(3.57%)	7(8.14%)	4(1.80%)		
T4	20(2.55%)	10(3.95%)	10(1.88%)			16(3.35%)	8(4.79%)	8(2.58%)			4(1.30%)	2(2.33%)	2(0.90%)		
Pathological N stage				5.753	0.124				2.557	0.465				2.639	0.451
N0	326(41.53%)	99(39.13%)	227(42.67%)			176(36.90%)	60(35.93%)	116(37.42%)			150(48.70%)	39(45.35%)	111(50.00%)		
N1	175(22.29%)	48(18.97%)	127(23.87%)			101(21.17%)	30(17.96%)	71(22.90%)			74(24.03%)	18(20.93%)	56(25.23%)		
N2	122(15.54%)	46(18.18%)	76(14.29%)			77(16.14%)	31(18.56%)	46(14.84%)			45(14.61%)	15(17.44%)	30(13.51%)		
N3	162(20.64%)	60(23.72%)	102(19.17%)			123(25.79%)	46(27.55%)	77(24.84%)			39(12.66%)	14(16.28%)	25(11.26%)		
Pathological TNM stage				9.303	0.026				6.677	0.083				4.817	0.186
Tis/T0	74(9.43%)	26(10.28%)	48(9.02%)			71(14.88%)	25(14.97%)	46(14.84%)			3(0.97%)	1(1.16%)	2(0.90%)		
I	157(20.00%)	47(18.58%)	110(20.68%)			83(17.40%)	32(19.16%)	51(16.45%)			74(24.03%)	15(17.44%)	59(26.58%)		
II	262(33.38%)	69(27.27%)	193(36.28%)			118(24.74%)	30(17.96%)	88(28.39%)			144(46.75%)	39(45.35%)	105(47.30%)		
III	292(37.20%)	111(43.87%)	181(34.02%)			205(42.98%)	80(47.91%)	125(40.32%)			87(28.25%)	31(36.05%)	56(25.23%)		
Total lymph nodes				2.325	0.127				2.587	1.608				1.216	0.270
＜21	391(49.81%)	136(53.75%)	255(47.93%)			202(42.35%)	79(47.31%)	123(39.68%)			189(61.36%)	57(66.28%)	132(59.46%)		
⩾21	394(50.19%)	117(46.25%)	277(52.07%)			275(57.65%)	88(52.69%)	187(60.32%)			119(38.64%)	29(33.72%)	90(40.54%)		
Positive lymph nodes				0.872	0.350				0.280	0.597				0.229	0.632
＜1	329(41.91%)	100(39.53%)	229(43.05%)			179(37.53%)	60(35.93%)	119(38.39%)			150(48.70%)	40(46.51%)	110(49.55%)		

⩾1	456(58.09%)	153(60.47%)	303(56.95%)			298(62.47%)	107(64.07%)	191(61.61%)			158(51.30%)	46(53.49%)	112(50.45%)		
Total axillary lymph nodes				0.500	0.480				1.726	0.189				0.048	0.827
＜20	389(49.55%)	130(51.38%)	259(48.68%)			195(40.88%)	75(44.91%)	120(38.71%)			194(62.99%)	55(63.95%)	139(62.61%)		
⩾20	396(50.45%)	123(48.62%)	273(51.32%)			282(59.12%)	92(55.09%)	190(61.29%)			114(37.01%)	31(36.05%)	83(37.39%)		
Positive axillary lymph nodes				2.160	0.142				0.289	0.591				1.587	0.208
＜1	343(43.69%)	101(39.92%)	242(45.49%)			182(38.16%)	61(36.53%)	121(39.03%)			161(52.27%)	40(46.51%)	121(54.50%)		
⩾1	442(56.31%)	152(60.08%)	290(54.51%)			295(61.84%)	106(63.47%)	189(60.97%)			147(47.73%)	46(53.49%)	101(45.50%)		
Postoperative complications				0.012	0.913				0.007	0.936				0.002	0.966
No	728(92.74%)	235(92.89%)	493(92.67%)			449(94.13%)	157(94.01%)	292(94.19%)			279(90.58%)	78(90.70%)	201(90.54%)		
Yes	57(7.26%)	18(7.11%)	39(7.33%)			28(5.87%)	10(5.99%)	18(5.81%)			29(9.42%)	8(9.30%)	21(9.46%)		
Postoperative chemotherapy				1.029	0.311				0.235	0.628				1.117	0.291
No	269(34.27%)	93(36.76%)	176(33.08%)			247(51.78%)	89(53.29%)	158(50.97%)			22(7.14%)	4(4.65%)	18(8.11%)		
Yes	516(65.73%)	160(63.24%)	356(66.92%)			230(48.22%)	78(46.71%)	152(49.03%)			286(92.86%)	82(95.35%)	204(91.89%)		
Postoperative chemotherapy times				3.072	0.080				2.183	0.140				2.005	0.157
＜4	374(47.64%)	132(52.17%)	242(45.49%)			340(71.28%)	126(75.45%)	214(69.03%)			34(11.04%)	6(6.98%)	28(12.61%)		
⩾4	411(52.36%)	121(47.83%)	290(54.51%)			137(28.72%)	41(24.55%)	96(30.97%)			274(88.96%)	80(93.02%)	194(87.39%)		
Postoperative radiotherapy				0.726	0.394				0.088	0.767				1.054	0.305
No	196(24.97%)	68(26.88%)	128(24.06%)			119(24.95%)	43(25.75%)	76(24.52%)			77(25.00%)	25(29.07%)	52(23.42%)		
Yes	589(75.03%)	185(73.12%)	404(75.94%)			358(75.05%)	124(74.25%)	234(75.48%)			231(75.00%)	61(70.93%)	170(76.58%)		
Postoperative endocrine therapy				0.274	0.601				0.169	0.681				0.592	0.442
No	302(38.47%)	94(37.15%)	208(39.10%)			206(43.19%)	70(41.92%)	136(43.87%)			96(31.17%)	24(27.91%)	72(32.43%)		
Yes	483(61.53%)	159(62.85%)	324(60.90%)			271(56.81%)	97(58.08%)	174(56.13%)			212(68.83%)	62(72.09%)	150(67.57%)		
Postoperative targeted therapy				1.137	0.286				1.993	0.158				0.090	0.765
No	583(74.27%)	194(76.68%)	389(73.12%)			332(69.60%)	123(73.65%)	209(67.42%)			251(81.49%)	71(82.56%)	180(81.08%)		
Yes	202(25.73%)	59(23.32%)	143(26.88%)			145(30.40%)	44(26.35%)	101(32.58%)			57(18.51%)	15(17.44%)	42(18.92%)		
