# Supplementary material for: Prognostic Nutritional Index (PNI) in Patients With Breast Cancer Treated With Neoadjuvant Chemotherapy as a Useful Prognostic Indicator
Source: Front Cell Dev Biol. 2021 Mar 30;9:656741. doi: 10.3389/fcell.2021.656741 (PMC8042235; doi:10.3389/fcell.2021.656741)
Supplement: Supplementary file 2 [file Table_2.pdf]

Supplementary Table 2 The correlations between nutritional parameters / blood parameters and PNI

Parameters	N	PNI 785				N	PNI 477				N	PNI 308			
Cases (n)	785	Low PNI 253	High PNI 532	χ2	P value		Low PNI 167	High PNI 310	χ2	P value		Low PNI 86	High PNI 222	χ2	P value
ALT (U/L)				0.356	0.551				0.379	0.538				0.236	0.627
＜15	370(47.13%)	123(48.62%)	247(46.43%)			208(43.61%)	76(45.51%)	132(42.58%)			162(52.60%)	47(54.65%)	115(51.80%)		
⩾15	416(52.99%)	130(51.38%)	286(53.76%)			269(56.39%)	91(54.49%)	178(57.42%)			147(47.73%)	39(45.35%)	108(48.65%)		
AST (U/L)				0.890	0.345				0.048	0.827				2.637	0.104
＜18	378(48.15%)	128(50.59%)	250(46.99%)			211(44.23%)	75(44.91%)	136(43.87%)			167(54.22%)	53(61.63%)	114(51.35%)		
⩾18	407(51.85%)	125(49.41%)	282(53.01%)			266(55.77%)	92(55.09%)	174(56.13%)			141(45.78%)	33(38.37%)	108(48.65%)		
LDH (U/L)				0.543	0.461				0.007	0.933				3.188	0.074
＜167	376(47.90%)	126(49.80%)	250(46.99%)			193(40.46%)	68(40.72%)	125(40.32%)			183(59.42%)	58(67.44%)	125(56.31%)		
⩾167	409(52.10%)	127(50.20%)	282(53.01%)			284(59.54%)	99(59.28%)	185(59.68%)			125(40.58%)	28(32.56%)	97(43.69%)		
GGT (U/L)				2.363	0.124				0.001	0.989				8.544	0.004
＜17	366(46.62%)	128(50.59%)	238(44.74%)			203(42.56%)	71(42.51%)	132(42.58%)			163(52.92%)	57(66.28%)	106(47.75%)		
⩾17	419(53.38%)	125(49.41%)	294(55.26%)			274(57.44%)	96(57.49%)	178(57.42%)			145(47.08%)	29(33.72%)	116(52.25%)		
ALP (U/L)				0.706	0.401				0.236	0.627				0.627	0.428
＜64	377(48.03%)	127(50.20%)	250(46.99%)			227(47.59%)	82(49.10%)	145(46.77%)			150(48.70%)	45(52.33%)	105(47.30%)		
⩾64	408(51.97%)	126(49.80%)	282(53.01%)			250(52.41%)	85(50.90%)	165(53.23%)			158(51.30%)	41(47.67%)	117(52.70%)		
GLU (mmol/L)				0.024	0.877				0.755	0.385				2.497	0.114
＜5.33	391(49.81%)	125(49.41%)	266(50.00%)			247(51.78%)	91(54.49%)	156(50.32%)			144(46.75%)	34(39.53%)	110(49.55%)		
⩾5.33	394(50.19%)	128(50.59%)	266(50.00%)			230(48.22%)	76(45.51%)	154(49.68%)			164(53.25%)	52(60.47%)	112(50.45%)		
IgA (g/L)				0.572	0.500				0.894	0.344				0.015	0.901
＜2.30	388(49.43%)	130(51.38%)	258(48.50%)			243(50.94%)	90(53.89%)	153(49.35%)			145(47.08%)	40(46.51%)	105(47.30%)		
⩾2.30	397(50.57%)	123(48.62%)	274(51.50%)			234(49.06%)	77(46.11%)	157(50.65%)			163(52.92%)	46(53.49%)	117(52.70%)		
IgG (g/L)				5.675	0.017				3.276	0.070				2.544	0.111
＜11.70	386(49.17%)	140(55.34%)	246(46.24%)			233(48.85%)	91(54.49%)	142(45.81%)			153(49.68%)	49(56.98%)	104(46.85%)		
⩾11.70	399(50.83%)	113(44.66%)	286(53.76%)			244(51.15%)	76(45.51%)	168(54.19%)			155(50.32%)	37(43.02%)	118(53.15%)		
IgM (g/L)				0.038	0.846				3.380	0.066				5.171	0.023

＜1.10	387(49.30%)	126(49.80%)	261(49.06%)			244(51.15%)	95(56.89%)	149(48.06%)			143(46.43%)	31(36.05%)	112(50.45%)		
⩾1.10	398(50.70%)	127(50.20%)	271(50.94%)			233(48.85%)	72(43.11%)	161(51.94%)			165(53.57%)	55(63.95%)	110(49.55%)		
ALB (g/L)				241.100	＜0.0001				184.400	＜0.0001				62.690	＜0.0001
＜45.2	392(49.94%)	228(90.12%)	164(30.83%)			235(49.27%)	153(91.62%)	82(26.45%)			157(50.97%)	75(87.21%)	82(36.94%)		
⩾45.2	393(50.06%)	25(9.88%)	368(69.17%)			242(50.73%)	14(8.38%)	228(73.55%)			151(49.03%)	11(12.79%)	140(63.06%)		
CRP (mg/dl)				2.703	0.100				9.251	0.002				4.472	0.035
＜0.2	384(48.92%)	113(44.66%)	271(50.94%)			187(39.20%)	50(29.94%)	137(44.19%)			197(63.96%)	63(73.26%)	134(60.36%)		
⩾0.2	401(51.08%)	140(55.34%)	261(49.06%)			290(60.80%)	117(70.06%)	173(55.81%)			111(36.04%)	23(26.74%)	88(39.64%)		
CA125 (U/ml)				0.128	0.721				0.005	0.943				0.036	0.849
＜13.35	392(49.94%)	124(49.01%)	268(50.38%)			221(46.33%)	77(46.11%)	144(46.45%)			171(55.52%)	47(54.65%)	124(55.86%)		
⩾13.35	393(50.06%)	129(50.99%)	264(49.62%)			256(53.67%)	90(53.89%)	166(53.55%)			137(44.48%)	39(45.35%)	98(44.14%)		
CA153 (U/ml)				0.937	0.333				1.270	0.260				0.177	0.674
＜11.63	392(49.94%)	120(47.43%)	272(51.13%)			208(43.61%)	67(40.12%)	141(45.48%)			184(59.74%)	53(61.63%)	131(59.01%)		
⩾11.63	393(50.06%)	133(52.57%)	260(48.87%)			269(56.39%)	100(59.88%)	169(54.52%)			124(40.26%)	33(38.37%)	91(40.99%)		
CEA (ng/ml)				0.064	0.780				0.056	0.813				1.493	0.222
＜1.66	392(49.94%)	128(50.59%)	264(49.62%)			212(44.44%)	73(43.71%)	139(44.84%)			180(58.44%)	55(63.95%)	125(56.31%)		
⩾1.66	393(50.06%)	125(49.41%)	268(50.38%)			265(55.56%)	94(56.29%)	171(55.16%)			128(41.56%)	31(36.05%)	97(43.69%)		
D-D (mg/L)				3.209	0.073				1.372	0.242				0.699	0.403
＜0.29	387(49.30%)	113(44.66%)	274(51.50%)			200(41.93%)	64(38.32%)	136(43.87%)			187(60.71%)	49(56.98%)	138(62.16%)		
⩾0.29	398(50.70%)	140(55.34%)	258(48.50%)			277(58.07%)	103(61.68%)	174(56.13%)			121(39.29%)	37(43.02%)	84(37.84%)		
FIB (g/L)				0.595	0.441				1.631	0.202				0.579	0.447
＜2.85	388(49.43%)	120(47.43%)	268(50.38%)			216(45.28%)	69(41.32%)	147(47.42%)			172(55.84%)	51(59.30%)	121(54.50%)		
⩾2.85	397(50.57%)	133(52.57%)	264(49.62%)			261(54.72%)	98(58.68%)	163(52.58%)			136(44.16%)	35(40.70%)	101(45.50%)		
INR (INR)				0.949	0.330				2.547	0.111				0.154	0.695
＜0.93	365(46.50%)	124(49.01%)	241(45.30%)			177(37.11%)	70(41.92%)	107(34.52%)			188(61.04%)	54(62.79%)	134(60.36%)		
⩾0.93	420(53.50%)	129(50.99%)	291(54.70%)			300(62.89%)	97(58.08%)	203(65.48%)			120(38.96%)	32(37.21%)	88(39.64%)		
FDP (ug/ml)				2.018	0.155				1.601	0.206				0.659	0.417
＜1.40	367(46.75%)	109(43.08%)	258(48.50%)			137(28.72%)	42(25.15%)	95(30.65%)			230(74.68%)	67(77.91%)	163(73.42%)		
⩾1.40	418(53.25%)	144(56.92%)	274(51.50%)			340(71.28%)	125(74.85%)	215(69.35%)			78(25.32%)	19(22.09%)	59(26.58%)		

White blood cell (W) ($\times 10^9$/L)				31.300	＜0.0001				25.540	＜0.0001				6.609	0.010
＜6.01	389(49.55%)	162(64.03%)	227(42.67%)			239(50.10%)	110(65.87%)	129(41.61%)			150(48.70%)	52(60.47%)	98(44.14%)		
⩾6.01	396(50.45%)	91(35.97%)	305(57.33%)			238(49.90%)	57(34.13%)	181(58.39%)			158(51.30%)	34(39.53%)	124(55.86%)		
Red blood cell (R) ($\times 10^{12}$/L)				26.380	＜0.0001				19.040	＜0.0001				7.808	0.005
＜4.40	389(49.55%)	159(62.85%)	230(43.23%)			235(49.27%)	105(62.87%)	130(41.94%)			154(50.00%)	54(62.79%)	100(45.05%)		
⩾4.40	396(50.45%)	94(37.15%)	302(56.77%)			242(50.73%)	62(37.13%)	180(58.06%)			154(50.00%)	32(37.21%)	122(54.95%)		
Hemoglobin (Hb) ($\times 10^9$/L)				37.140	＜0.0001				21.100	＜0.0001				15.030	0.0001
＜132	382(48.66%)	163(64.43%)	219(41.17%)			243(50.94%)	109(65.27%)	134(43.23%)			139(45.13%)	54(62.79%)	85(38.29%)		
⩾132	403(51.34%)	90(35.57%)	313(58.83%)			234(49.06%)	58(34.73%)	176(56.77%)			169(54.87%)	32(37.21%)	137(61.71%)		
Neutrophil (N) ($\times 10^9$/L)				8.124	0.004				7.056	0.008				1.949	0.163
＜3.68	392(49.94%)	145(57.31%)	247(46.43%)			229(48.01%)	94(56.29%)	135(43.55%)			163(52.92%)	51(59.30%)	112(50.45%)		
⩾3.68	393(50.06%)	108(42.69%)	285(53.57%)			248(51.99%)	73(43.71%)	175(56.45%)			145(47.08%)	35(40.70%)	110(49.55%)		
Lymphocyte (L) ($\times 10^9$/L)				19.600	＜0.0001				17.430	＜0.0001				2.261	0.133
＜1.76	391(49.81%)	155(61.26%)	236(44.36%)			258(54.09%)	112(67.07%)	146(47.10%)			133(43.18%)	43(50.00%)	90(40.54%)		
⩾1.76	394(50.19%)	98(38.74%)	296(55.64%)			219(45.91%)	55(32.93%)	164(52.90%)			175(56.82%)	43(50.00%)	132(59.46%)		
Monocyte (M) ($\times 10^9$/L)				5.788	0.016				1.512	0.219				6.248	0.012
＜0.35	367(46.75%)	134(52.96%)	233(43.80%)			216(45.28%)	82(49.10%)	134(43.23%)			151(49.03%)	52(60.47%)	99(44.59%)		
⩾0.35	418(53.25%)	119(47.04%)	299(56.20%)			261(54.72%)	85(50.90%)	176(56.77%)			157(50.97%)	34(39.53%)	123(55.41%)		
Eosinophils (E) ($\times 10^9$/L)				2.713	0.100				2.005	0.157				1.801	0.180
＜0.06	356(45.35%)	104(41.11%)	252(47.37%)			241(50.52%)	77(46.11%)	164(52.90%)			115(37.34%)	27(31.40%)	88(39.64%)		
⩾0.06	429(54.65%)	149(58.89%)	280(52.63%)			236(49.48%)	90(53.89%)	146(47.10%)			193(62.66%)	59(68.60%)	134(60.36%)		
Basophils (B) ($\times 10^9$/L)				8.078	0.005				8.100	0.004				0.929	0.335
＜0.02	224(28.54%)	89(35.18%)	135(25.38%)			136(28.51%)	61(36.53%)	75(24.19%)			88(28.57%)	28(32.56%)	60(27.03%)		
⩾0.02	561(71.46%)	164(64.82%)	397(74.62%)			341(71.49%)	106(63.47%)	235(75.81%)			220(71.43%)	58(67.44%)	162(72.97%)		
Platelet (P) ($\times 10^9$/L)				11.240	0.001				8.975	0.003				3.367	0.067
＜243	388(49.43%)	147(58.10%)	241(45.30%)			224(46.96%)	94(56.29%)	130(41.94%)			164(53.25%)	53(61.63%)	111(50.00%)		
⩾243	397(50.57%)	106(41.90%)	291(54.70%)			253(53.04%)	73(43.71%)	180(58.06%)			144(46.75%)	33(38.37%)	111(50.00%)		
