# Supplementary material for: Prognostic Nutritional Index (PNI) in Patients With Breast Cancer Treated With Neoadjuvant Chemotherapy as a Useful Prognostic Indicator
Source: Front Cell Dev Biol. 2021 Mar 30;9:656741. doi: 10.3389/fcell.2021.656741 (PMC8042235; doi:10.3389/fcell.2021.656741)
Supplement: Supplementary file 3 [file Table_3.pdf]

Supplementary Table 3 Univariate and multivariate Cox regression survival analyses of the SII for the prediction of DFS and OS in breast cancer patients

		DFS				OS		
	Univariate analysis		Multivariate analysis		Univariate analysis		Multivariate analysis	
Parameters	Hazard ratio (95%CI)	P value	Hazard ratio (95%CI)	P value	Hazard ratio (95%CI)	P value	Hazard ratio (95%CI)	P value
Age (year)		0.539				0.975		
< 47	1(reference)				1(reference)			
⩾47	0.897(0.636-1.266)				0.994(0.703-1.406)			
Marital status		0.554				0.577		
Married	1(reference)				1(reference)			
Unmarried	0.801(0.384-1.667)				0.808(0.383-1.704)			
Occupation		0.862				0.497		
Mental worker	1(reference)				1(reference)			
Manual worker	1.023(0.715-1.463)				0.855(0.598-1.223)			
Others	1.079(0.818-1.423)				1.077(0.812-1.429)			
Weight (Kg)		0.106				0.192		
< 62.00	1(reference)				1(reference)			
⩾62.00	1.372(0.934-2.017)				1.304(0.874-1.946)			
Height (m)		0.584				0.658		
< 1.60	1(reference)				1(reference)			
⩾1.60	0.925(0.700-1.221)				0.937(0.705-1.246)			
BMI		0.371				0.781		
< 24.00	1(reference)				1(reference)			
⩾24.00	0.837(0.567-1.235)				0.944(0.631-1.412)			
Family history		0.443				0.982		
No	1(reference)				1(reference)			
Yes	0.890(0.661-1.197)				1.003(0.742-1.356)			
Menarche age (year)		0.611				0.269		

< 14	1(reference)				1(reference)			
⩾14	1.067(0.829-1.374)				1.159(0.891-1.508)			
Menopause		0.008		0.002		< 0.0001		0.006
No	1(reference)		1(reference)		1(reference)		1(reference)	
Yes	1.637(1.137-2.357)		1.439(1.147-1.806)		1.552(1.217-1.979)		1.380(1.098-1.733)	
ALT (U/L)		0.470				0.070		
< 15	1(reference)				1(reference)			
⩾15	0.892(0.656-1.214)				0.746(0.543-1.024)			
AST (U/L)		0.604				0.925		
< 18	1(reference)				1(reference)			
⩾18	0.920(0.672-1.259)				1.015(0.737-1.397)			
LDH (U/L)		0.483				0.901		
< 167	1(reference)				1(reference)			
⩾167	1.106(0.834-1.466)				1.018(0.766-1.352)			
GGT (U/L)		0.683				0.568		
< 17	1(reference)				1(reference)			
⩾17	1.061(0.797-1.412)				1.089(0.811-1.463)			
ALP (U/L)		0.081				0.111		
< 64	1(reference)				1(reference)			
⩾64	1.288(0.969-1.712)				1.262(0.947-1.682)			
GLU (mmol/L)		0.003		0.002		0.013		0.023
< 5.33	1(reference)		1(reference)		1(reference)		1(reference)	
⩾5.33	0.658(0.500-0.864)		0.700(0.559-0.878)		0.695(0.522-0.927)		0.756(0.594-0.961)	
IgA (g/L)		0.853				0.372		
< 2.30	1(reference)				1(reference)			
⩾2.30	1.024(0.794-1.321)				1.124(0.868-1.456)			
IgG (g/L)		0.908				0.967		
< 11.70	1(reference)				1(reference)			
⩾11.70	1.014(0.790-1.302)				1.005(0.782-1.291)			

IgM (g/L)		0.401				0.569		
< 1.10	1(reference)				1(reference)			
⩾1.10	0.898(0.700-1.152)				0.928(0.719-1.198)			
ALB (g/L)		0.050				0.439		
< 45.2	1(reference)				1(reference)			
⩾45.2	1.260(1.000-1.587)				1.105(0.856-1.426)			
CRP (mg/dl)		0.330				0.736		
< 0.2	1(reference)				1(reference)			
⩾0.2	0.868(0.653-1.153)				0.949(0.704-1.280)			
CA125 (U/ml)		0.058				0.201		
< 13.35	1(reference)				1(reference)			
⩾13.35	1.266(0.992-1.616)				1.186(0.912-1.542)			
CA153 (U/ml)		0.104				0.002		0.001
< 11.63	1(reference)				1(reference)		1(reference)	
⩾11.63	1.260(0.953-1.665)				1.553(1.171-2.061)		1.438(1.153-1.793)	
CEA (ng/ml)		0.507				0.841		
< 1.66	1(reference)				1(reference)			
⩾1.66	0.915(0.706-1.187)				1.027(0.788-1.338)			
D-D (mg/L)		0.164				0.240		
< 0.29	1(reference)				1(reference)			
⩾0.29	1.215(0.923-1.600)				1.183(0.893-1.567)			
FIB (g/L)		0.523				0.256		
< 2.85	1(reference)				1(reference)			
⩾2.85	0.916(0.701-1.197)				1.167(0.893-1.525)			
INR		0.707				0.048		0.013
< 0.93	1(reference)				1(reference)		1(reference)	
⩾0.93	0.953(0.741-1.224)				1.292(1.002-1.666)		1.322(1.061-1.649)	
FDP (ug/ml)		0.037		0.027		0.334		
< 1.40	1(reference)		1(reference)		1(reference)			

⩾1.40	1.307(1.017-1.681)		1.301(1.030-1.643)		0.861(0.635-1.166)			
ABO blood type		0.087				0.079		
A	1(reference)				1(reference)			
B	0.919(0.674-1.253)				0.854(0.618-1.180)			
O	0.733(0.529-1.015)				0.768(0.548-1.076)			
AB	1.248(0.795-1.958)				1.355(0.848-2.165)			
White blood cell (W)×10^9/L		0.067				0.127		
＜6.01	1(reference)				1(reference)			
⩾6.01	1.457(0.974-2.181)				1.386(0.911-2.108)			
Red blood cell (R)×10^{12}/L		0.859				0.399		
＜4.40	1(reference)				1(reference)			
⩾4.40	0.972(0.716-1.320)				1.143(0.837-1.560)			
Hemoglobin (Hb)×10^9/L		0.778				0.355		
＜132	1(reference)				1(reference)			
⩾132	0.957(0.707-1.295)				0.868(0.644-1.170)			
Neutrophil (N)×10^9/L		0.119				0.162		
＜3.68	1(reference)				1(reference)			
⩾3.68	0.732(0.495-1.083)				0.751(0.504-1.121)			
Lymphocyte (L)×10^9/L		0.913				0.033		0.029
＜1.76	1(reference)				1(reference)		1(reference)	
⩾1.76	1.017(0.749-1.379)				1.325(1.023-1.716)		1.309(1.028-1.666)	
Monocyte (M)×10^9/L		0.340				0.005		0.002
＜0.35	1(reference)				1(reference)		1(reference)	
⩾0.35	0.877(0.671-1.147)				0.672(0.510-0.885)		0.705(0.564-0.882)	
Eosinophils (E)×10^9/L		0.036		0.029		0.093		
＜0.06	1(reference)		1(reference)		1(reference)			
⩾0.06	0.752(0.577-0.981)		0.780(0.624-0.974)		0.812(0.637-1.035)			
Basophils (B)×10^9/L		0.828				0.517		

< 0.02	1(reference)				1(reference)			
⩾0.02	1.033(0.769-1.387)				1.103(0.819-1.485)			
Platelet (P)×10^9/L		0.003		0.006		0.061		
< 243	1(reference)		1(reference)		1(reference)			
⩾243	0.666(0.510-0.869)		0.704(0.550-0.902)		0.776(0.595-1.011)			
Systemic immune-inflammation index (SII)		< 0.0001		< 0.0001		< 0.0001		< 0.0001
< 560	1(reference)		1(reference)		1(reference)		1(reference)	
⩾560	1.965(1.430-2.701)		1.676(1.334-2.105)		2.086(1.518-2.868)		2.073(1.640-2.622)	
Tumor site		0.205				0.279		
Right	1(reference)				1(reference)			
Left	1.174(0.915-1.506)				1.149(0.893-1.477)			
US-Primary tumor site		0.429				0.548		
Upper outer quadrant	1(reference)				1(reference)			
Lower outer quadrant	1.318(0.889-1.956)				1.294(0.859-1.950)			
Lower inner quadrant	1.115(0.644-1.930)				1.476(0.853-2.553)			
Upper inner quadrant	1.274(0.910-1.785)				1.111(0.782-1.578)			
Central	1.349(0.768-2.371)				1.093(0.619-1.929)			
US-Tumor size (cm)		0.389				0.639		
⩽2cm	1(reference)				1(reference)			
> 2 and < 5cm	0.823(0.602-1.126)				0.921(0.670-1.266)			
⩾5cm	1.002(0.543-1.848)				0.739(0.394-1.384)			
US-LNM		0.861				0.477		
No	1(reference)				1(reference)			
Yes	0.969(0.681-1.377)				1.138(0.796-1.625)			
US-BIRADS		0.676				0.509		
4 (4a 4b 4c)	1(reference)				1(reference)			
5	0.812(0.505-1.304)				0.766(0.457-1.284)			
6	0.857(0.528-1.390)				0.849(0.500-1.441)			
Clinical stage								

Clinical T stage		0.031		0.022		0.023		0.011
T1	1(reference)		1(reference)		1(reference)		1(reference)	
T2	2.323(1.332-4.051)		1.930(1.183-3.150)		2.413(1.345-4.328)		2.305(1.392-3.817)	
T3	2.228(1.112-4.463)		2.004(1.103-3.641)		2.698(1.320-5.510)		2.426(1.315-4.476)	
T4	2.121(1.055-4.745)		1.851(1.072-3.671)		2.861(1.236-6.619)		2.333(1.158-4.703)	
Clinical N stage		0.188				0.320		
N0	1(reference)				1(reference)			
N1	0.960(0.640-1.439)				1.029(0.666-1.590)			
N2	1.004(0.501-2.015)				0.992(0.486-2.028)			
N3	1.684(0.786-3.608)				1.626(0.723-3.656)			
Clinical TNM stage		0.185				0.417		
I	1(reference)				1(reference)			
II	0.563(0.300-1.055)				0.641(0.329-1.247)			
III	0.630(0.259-1.530)				0.666(0.258-1.718)			
Operative time (min)		0.292				0.121		
＜90	1(reference)				1(reference)			
⩾90	0.859(0.649-1.138)				0.816(0.631-1.055)			
Type of surgery		0.221				0.316		
Mastectomy	1(reference)				1(reference)			
Breast-conserving surgery	0.799(0.558-1.144)				1.208(0.834-1.748)			
Histologic type		0.020		0.027		0.001		0.011
Ductal	1(reference)		1(reference)		1(reference)		1(reference)	
Lobular	2.518(1.102-5.750)		2.473(1.131-5.410)		3.314(1.394-7.877)		2.140(1.035-4.423)	
Others	2.118(1.044-4.680)		1.798(1.159-3.749)		3.136(1.417-6.940)		2.312(1.125-4.752)	
Histologic grade		0.120				0.203		
I	1(reference)				1(reference)			
II	0.820(0.514-1.308)				0.810(0.506-1.296)			
III	0.643(0.392-1.056)				0.658(0.394-1.098)			
Pathological TNM classification								

Pathological T stage		0.125				0.052		
Tis/T0	1(reference)				1(reference)			
T1	0.734(0.239-2.248)				0.594(0.195-1.808)			
T2	0.708(0.225-2.224)				0.480(0.153-1.504)			
T3	0.600(0.167-2.145)				0.379(0.107-1.342)			
T4	1.703(0.430-6.731)				1.198(0.305-4.706)			
Pathological N stage		0.016		0.048		0.002		0.001
N0	1(reference)		1(reference)		1(reference)		1(reference)	
N1	2.638(1.074-7.476)		1.402(1.039-1.892)		1.236(1.171-1.790)		1.283(1.041-1.713)	
N2	2.911(1.294-10.95)		1.149(1.086-1.649)		2.600(1.245-5.429)		1.413(1.003-1.991)	
N3	4.740(1.264-17.77)		1.522(1.078-2.150)		4.241(1.929-9.325)		1.880(1.371-2.578)	
Pathological TNM stage		0.284				0.018		0.012
Tis/T0	1(reference)				1(reference)		1(reference)	
I	3.206(0.885-11.61)				3.027(1.202-11.01)		2.118(1.565-7.025)	
II	3.503(0.925-13.25)				3.832(1.009-14.55)		2.429(1.393-8.223)	
III	2.660(0.554-12.75)				2.532(1.337-4.796)		2.645(1.428-4.899)	
Total lymph nodes		0.727				0.543		
＜21	1(reference)				1(reference)			
⩾21	0.906(0.521-1.575)				0.832(0.461-1.502)			
Positive lymph nodes		0.314				0.923		
＜1	1(reference)				1(reference)			
⩾1	0.526(0.151-1.835)				0.938(0.256-3.437)			
Total axillary lymph nodes		0.329				0.528		
＜20	1(reference)				1(reference)			
⩾20	0.760(0.438-1.318)				0.827(0.460-1.489)			
Positive axillary lymph nodes		0.824				0.671		
＜1	1(reference)				1(reference)			
⩾1	0.904(0.374-2.183)				0.815(0.318-2.088)			
Postoperative pathology (IHC)								

Molecular subtype		0.039		0.009		0.114		
Luminal A	1(reference)		1(reference)		1(reference)			
Luminal B HER2+	0.248(0.092-0.670)		0.372(0.199-0.697)		0.290(0.105-0.800)			
Luminal B HER2-	0.608(0.354-0.958)		0.504(0.305-0.830)		0.533(0.308-0.921)			
HER2 enriched	0.186(0.064-0.546)		0.325(0.174-0.609)		0.358(0.120-1.069)			
Triple negative	0.591(0.294-0.843)		0.527(0.300-0.924)		0.658(0.328-1.321)			
ER status		0.087				0.940		
Negative	1(reference)				1(reference)			
Positive	0.642(0.386-1.066)				0.980(0.593-1.620)			
PR status		0.256				0.217		
Negative	1(reference)				1(reference)			
Positive	1.256(0.847-1.862)				1.265(0.870-1.838)			
HER2 status		0.111				0.204		
Negative (0--++)	1(reference)				1(reference)			
Positive (+++)	2.061(0.846-5.025)				1.769(0.732-4.271)			
Ki-67 status		0.003		0.001		0.006		0.001
Negative (≤14%)	1(reference)		1(reference)		1(reference)		1(reference)	
Positive (> 14%)	1.694(1.197-2.396)		1.666(1.222-2.272)		1.631(1.154-2.306)		1.691(1.251-2.286)	
AR status		0.474				0.820		
Negative	1(reference)				1(reference)			
Positive	0.848(0.541-1.330)				0.943(0.574-1.551)			
CK5/6 status		0.024		0.0001		0.049		0.0002
Negative	1(reference)		1(reference)		1(reference)		1(reference)	
Positive	1.674(1.071-2.614)		1.900(1.371-2.632)		1.600(1.001-2.557)		1.847(1.335-2.556)	
E-cad status		0.173				< 0.0001		< 0.0001
Negative	1(reference)				1(reference)		1(reference)	
Positive	1.271(0.899-1.797)				2.775(1.895-4.066)		2.821(2.124-3.747)	
EGFR status		0.313				0.932		
Negative	1(reference)				1(reference)			

Positive	0.811(0.540-1.217)				0.981(0.636-1.512)			
P53 status		0.071				0.092		
Negative	1(reference)				1(reference)			
Positive	0.773(0.585-1.021)				0.815(0.643-1.034)			
TOP2A status		0.292				0.479		
Negative	1(reference)				1(reference)			
Positive	1.212(0.847-1.734)				1.146(0.785-1.672)			
Lymph vessel invasion		0.028		0.022		0.016		0.002
Negative	1(reference)		1(reference)		1(reference)		1(reference)	
Positive	1.433(1.040-1.977)		1.358(1.044-1.765)		1.492(1.077-2.068)		1.481(1.152-1.904)	
Neural invasion		0.577				0.755		
Negative	1(reference)				1(reference)			
Positive	0.887(0.584-1.348)				1.066(0.710-1.601)			
Postoperative chemotherapy		< 0.0001		< 0.0001		0.0001		0.0001
No	1(reference)		1(reference)		1(reference)		1(reference)	
Yes	2.108(1.436-3.094)		1.807(1.297-2.518)		1.931(1.311-2.843)		1.692(1.235-2.318)	
Postoperative radiotherapy		0.281				0.164		
No	1(reference)				1(reference)			
Yes	1.198(0.862-1.665)				1.273(0.906-1.789)			
Postoperative endocrine therapy		0.051				0.211		
No	1(reference)				1(reference)			
Yes	1.366(0.999-1.867)				1.217(0.894-1.657)			
Postoperative targeted therapy		< 0.0001		< 0.0001		0.013		< 0.0001
No	1(reference)		1(reference)		1(reference)		1(reference)	
Yes	2.480(1.713-3.591)		2.050(1.600-2.628)		1.592(1.105-2.293)		1.687(1.317-2.161)	
